# Analysis of the retinal gene expression profile after hypoxic preconditioning identifies candidate genes for neuroprotection

**DOI:** 10.1186/1471-2164-9-73

**Published:** 2008-02-08

**Authors:** Markus Thiersch, Wolfgang Raffelsberger, Rico Frigg, Marijana Samardzija, Andreas Wenzel, Olivier Poch, Christian Grimm

**Affiliations:** 1 Lab of Retinal Cell Biology, Dept Ophthalmology, University of Zurich, Switzerland; 2 Laboratoire de BioInformatique et Génomique Intégrative, Institut de Genetique et de Biologie Moleculaire et Cellulaire, 67404 Illkirch, France

## Abstract

**Background:**

Retinal degeneration is a main cause of blindness in humans. Neuroprotective therapies may be used to rescue retinal cells and preserve vision. Hypoxic preconditioning stabilizes the transcription factor HIF-1α in the retina and strongly protects photoreceptors in an animal model of light-induced retinal degeneration. To address the molecular mechanisms of the protection, we analyzed the transcriptome of the hypoxic retina using microarrays and real-time PCR.

**Results:**

Hypoxic exposure induced a marked alteration in the retinal transcriptome with significantly different expression levels of 431 genes immediately after hypoxic exposure. The normal expression profile was restored within 16 hours of reoxygenation. Among the differentially regulated genes, several candidates for neuroprotection were identified like metallothionein-1 and -2, the HIF-1 target gene adrenomedullin and the gene encoding the antioxidative and cytoprotective enzyme paraoxonase 1 which was previously not known to be a hypoxia responsive gene in the retina. The strongly upregulated cyclin dependent kinase inhibitor *p21 *was excluded from being essential for neuroprotection.

**Conclusion:**

Our data suggest that neuroprotection after hypoxic preconditioning is the result of the differential expression of a multitude of genes which may act in concert to protect visual cells against a toxic insult.

## Background

Retinal blinding diseases like retinitis pigmentosa (RP) and age related macular degeneration (AMD) are characterized by a progressive retinal degeneration which involves the apoptotic loss of photoreceptor cells. Although significant progress in the understanding of the molecular mechanisms leading to AMD and RP has been made in recent years, efficient treatments to successfully prevent loss of vision are still not available.

Neuroprotection is a strategy to preserve retinal function. It aims at the interference with regulatory mechanisms of cell death to protect photoreceptor cells. To successfully target these mechanisms it is necessary to understand the molecular signalling networks in the degenerating retina. Since neither an extrinsic (activation of caspases via death receptors) nor an intrinsic death pathway (release of cytochrome c from mitochondria) seems to be activated during retinal degeneration [1], mechanisms of photoreceptor cell death are still poorly understood. Several models of inherited [2] and induced [3] retinal degeneration are used to study the molecular events of photoreceptor apoptosis. Inherited models mostly show a slow progression of retinal degeneration resulting in constant but low levels of apoptosis. Models of induced retinal degeneration, like the light damage model [3], are easy to handle and the synchronized response to the apoptotic stimulus may raise apoptotic factors above detection threshold allowing their detailed investigation.

Various preconditioning protocols are used as a strategy to protect tissues from degenerative processes. Especially ischemic and hypoxic preconditioning successfully reduced the severity of induced or inherited degeneration in tissues like brain [4,5] heart [6,7] and the retina [8-12]. Hypoxia describes a state of low oxygen. It appears pathologically during several diseases like cancer, stroke or heart infarction [13] but also physiologically during development in many tissues [14]. In the adult retina, increased oxygen consumption during night time leads to borderline hypoxic conditions [15]. To cope with the reduced oxygen availability cells differentially regulate genes including factors involved in an anti-apoptotic response [16,17]. A key regulator of the tissue response to hypoxia is the transcription factor hypoxia inducible factor 1 (HIF-1), a heterodimeric protein consisting of the constitutively and stably expressed hypoxia inducible factor 1β (HIF-1β) and oxygen regulated subunit hypoxia inducible factor 1α (HIF-1α). During hypoxia HIF-1α is stabilized, enters the nucleus, recruits HIF-1β and regulates the expression of target genes involved in different pathways like apoptosis, metabolism or angiogenesis [18].

Hypoxic preconditioning was shown to stabilize HIF-1α in the retina [9,12]. Stabilization of this transcription factor induces the expression of target genes with neuroprotective properties like vascular endothelial growth factor (*Vegf*) and erythropoietin (*Epo*) suggesting a link between HIF-1 driven gene expression and neuroprotection [9,12]. Exogenous application of Epo not only protects retinal ganglion cells in a model of ischemia-reperfusion injury [19] but also photoreceptors in the model of light induced retinal degeneration [20,21]. However, protection of visual cells from light damage was weaker than after hypoxic preconditioning suggesting that factors in addition to Epo contribute to retinal protection by hypoxia. The identification of these factors is essential for the development of efficient neuroprotective strategies focused on the prevention of retinal degeneration.

We used whole genome microarrays and real-time PCR to detect expression of differentially regulated genes after hypoxic preconditioning in adult mouse retinas. The analysis of the hypoxic transcriptome characterized the response of the retina to low oxygen levels. Cyclin-dependent kinase inhibitor 1a (*p21*) was among the most strongly induced genes and occupied a central position in a differentially regulated gene network affecting cellular growth and proliferation. Using p21 gene knockout animals, we analyzed the impact of this gene on retinal neuroprotection in the model of light induced retinal degeneration.

## Results

### Time frame of neuroprotection after hypoxic preconditioning

**Figure 1:**
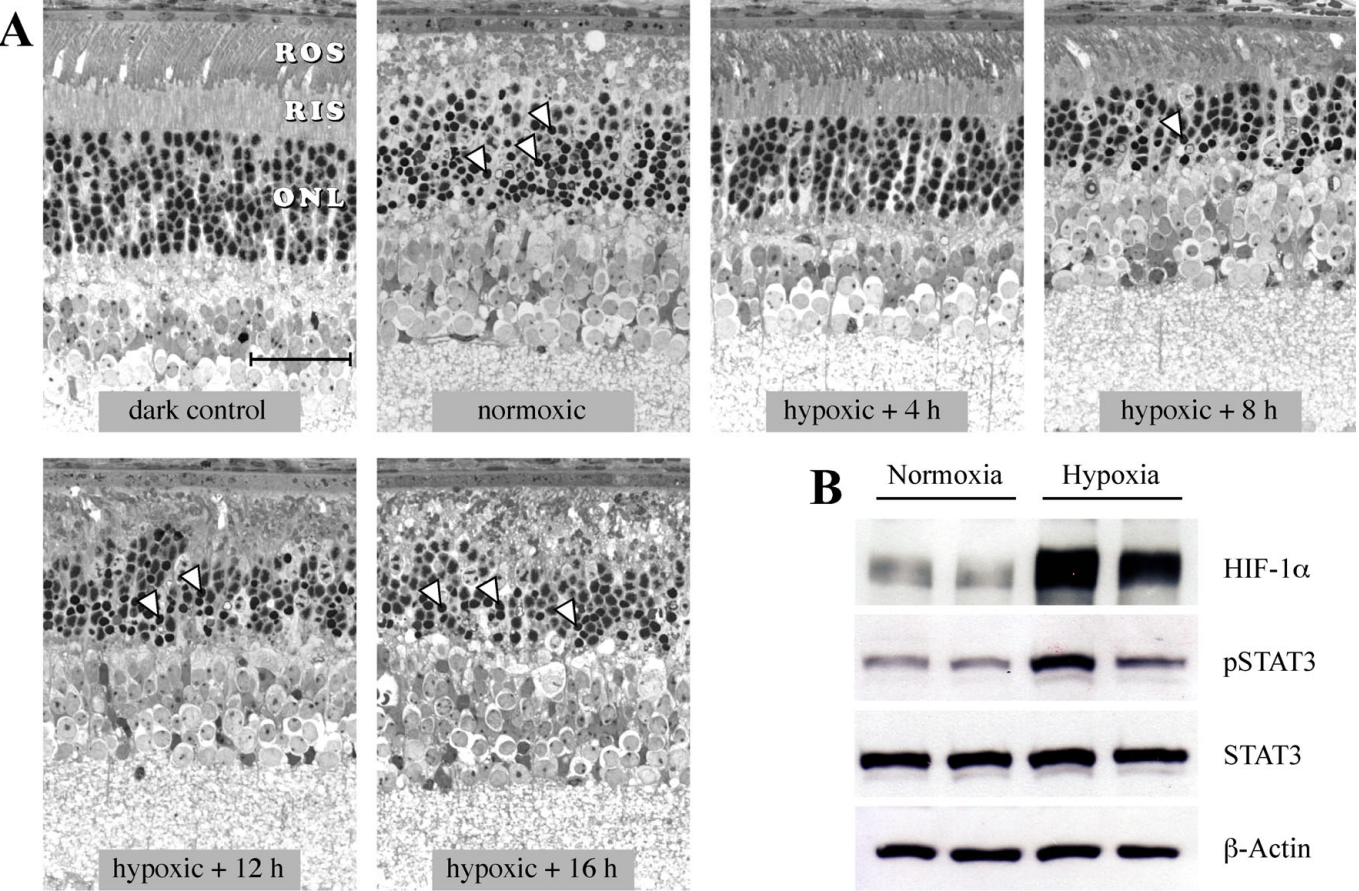
**Transient protection of retinal morphology by hypoxic preconditioning**. **A) **BALB/c mice were preconditioned by hypoxia for 6 h. After 4 h, 8 h, 12 h or 16 h of reoxygenation in darkness, mice were exposed to 5'000 lux of white light for 1 h. Control animals were not preconditioned and were (normoxic) or were not (dark control) exposed to light. Retinal morphology was analyzed 36 h after light exposure. Normoxic animals exposed to light showed a severe disruption of ROS and RIS with the appearance of many pycnotic photoreceptor nuclei. Mice exposed to light 4 hours after hypoxic preconditioning were almost completely protected and only some ROS vesiculation was observed. The protective effect of hypoxia was progressively weakened with increasing reoxygenation time before illumination as indicated by an increased disruption of ROS and RIS and the increased appearance of pycnotic photoreceptor nuclei. 16 h after preconditioning the protection was completely lost. Hypoxic preconditioning per se did not affect retinal morphology or function even after prolonged survival (data not shown, [9]). Arrowheads: examples of pycnotic nuclei; ROS: rod outer segments; RIS: rod inner segments; ONL: outer nuclear layer, scale bar: 25 μm. **B) **Expression of transcription factors HIF-1α and phospho-STAT3 in the retina was analyzed in normoxic mice or immediately after hypoxic preconditioning by Western blotting. β-actin and STAT3 levels verified equal sample loading. Both transcription factors were induced immediately after hypoxia with some variability between individuals. Shown are results from two normoxic controls and from two mice preconditioned with hypoxia.

In previous experiments we observed an almost complete protection of photoreceptors against light induced degeneration when exposed at 4 hours after hypoxic preconditioning. Protection, however, was lost after prolonged reoxygenation of 16 hours suggesting a rather short-lived neuroprotective effect of hypoxic preconditioning [9]. To analyze the time frame of hypoxia-induced neuroprotection in more detail, we allowed preconditioned mice to reoxygenate for 4 h, 8 h, 12 h and 16 h, respectively, before they were exposed to high levels of white light. As expected, retinal morphology (Fig. 1A) was almost completely preserved in mice illuminated after a reoxygenation period of 4 h. Only slight disturbances and vesiculations in the rod outer segments but no apoptotic nuclei with condensed chromatin were observed.

Exposure after 8 h of reoxygenation resulted in the appearance of some apoptotic photoreceptor nuclei. 12 h of reoxygenation further reduced the protection against light damage as evidenced by the appearance of many nuclei with condensed chromatin and an almost complete disintegration of rod inner (RIS) and rod outer segments (ROS). Retinas of mice illuminated 16 hours after hypoxic preconditioning were as susceptible to light damage as retinas of normoxic control mice (Fig. 1A).

### The retinal response to hypoxic preconditioning

It is well known that hypoxia alters the gene expression profile in a given tissue [22] in an attempt to cope with the unfavourable condition. One of the major factors regulating this response is the transcription factor HIF-1, which is activated in the hypoxic retina (Fig. 1B) [9]. Similarly, the pro-survival transcription factor Stat3 [23], which has been reported to be induced in several hypoxic tissues [24], was phosphorylated and thus activated in the hypoxic retina (Fig. 1B). The different levels of activation (shown are examples of two mice) point to a certain variability in the response to hypoxia between individual mice. Nevertheless, the activation of these transcription factors suggests a differential regulation of a multitude of potentially neuroprotective genes in the retina by hypoxic preconditioning. Based on the time frame of neuroprotection (Fig. 1A), we analyzed the gene expression pattern in the retina at 0 h, 2 h, 4 h, and at 16 h after hypoxia (see Methods).

Hierarchical clustering of gene chip data showed strong similarities of the three replica-chips of a respective time point after hypoxia [see additional file 1]. Such clustering was not observed in normoxic samples suggesting that hypoxia induced a strong and specific response in the retina. This hypoxic response quickly vanished and at 4 h after hypoxic preconditioning the retinal transcriptome was similar to normoxic controls.

**Figure 2:**
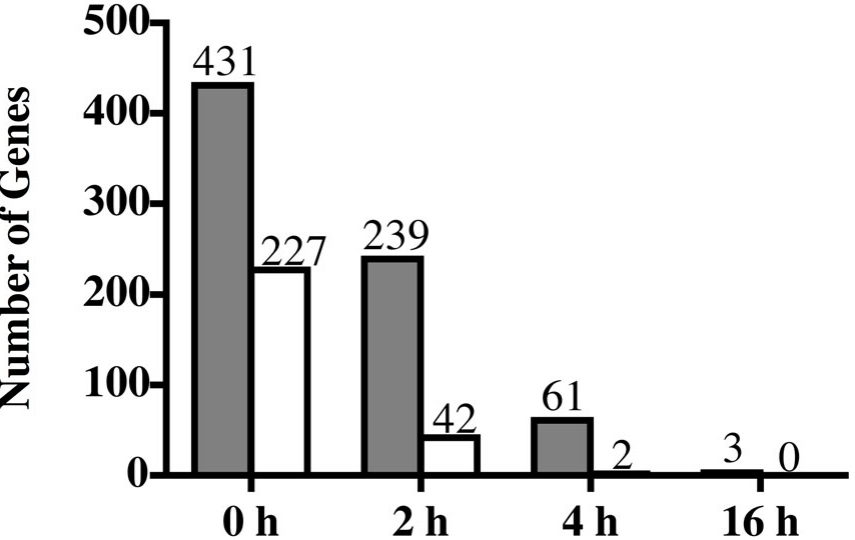
**Number of genes differentially expressed immediately (0 h), 2 h, 4 h and 16 h after hypoxic preconditioning**. Gray bars: total number of significantly regulated genes. White bars: number of genes regulated at least 2-fold. The number of differentially regulated genes decreased rapidly during reoxygenation, indicating a fast return to the gene expression pattern of normoxic retinas.

After normalization of the data by GCRMA algorithm to minimize the theoretical appearance of false positive signals we detected 431 differentially regulated genes immediately after hypoxia, 227 of which had a fold change above 2 (0 h, Fig. 2). The number of regulated genes decreased gradually until 16 h after hypoxia when just 3 genes showed altered expression levels (Fig. 2). The application of our statistical filter for a maximal acceptable FDR suggested that there are (statistically estimated) 25, 23, 6 and 1 false positive genes in the analyzed list of differentially regulated genes at 0 h, 2 h, 4 h and 16 h, respectively.

### Prominently regulated genes

The genes with the strongest regulation after hypoxic exposure are listed in Table 1 [for complete gene lists see additional file 2]. The most significantly upregulated gene (>118-fold) was found to be *Obox6*, a homeobox-containing, putative transcriptional activator of unknown function mainly expressed in oocytes [25]. The gene with the strongest downregulation was the cold-inducible RNA binding motif protein 3 (*Rbm3*) with a 19-fold reduced expression immediately after hypoxia (Table 1).

**Table 1:** Top 50 differentially regulated genes immediately after hypoxic preconditioning

**Affymetrix ID**	**Gene Symbol**	**Gene Name**	**FC**	**pVal**
UPREGULATED				

1440257_at	Obox6	oocyte specific homeobox 6	118.4	2.48E-07
1433837_at	8430408G22Rik	RIKEN cDNA 8430408G22 gene	35.6	6.94E-05
1424638_at	Cdkn1a	cyclin-dependent kinase inhibitor 1A (p21)	30.5	3.00E-05
1454409_at	4833408G04Rik	RIKEN cDNA 4833408G04 gene	24.0	2.07E-06
1418190_at	Pon1	paraoxonase 1	22.5	1.98E-05
1422832_at	Rgr	retinal G protein coupled receptor	22.1	6.20E-05
1454608_x_at	Ttr	transthyretin	20.1	2.67E-04
1455913_x_at	Ttr	transthyretin	20.0	3.11E-04
1458610_at	-	---	17.4	1.95E-04
1416434_at	Bcl2l10	Bcl2-like 10	17.0	2.70E-05
1444487_at	Lrat	lecithin-retinol acyltransferase	16.2	6.54E-06
1441228_at	Apold1	apolipoprotein L domain containing 1	11.7	7.21E-05
1438815_at	Hist2h2aa2	histone 2, H2aa2	10.7	1.01E-04
1416077_at	Adm	adrenomedullin	10.6	2.48E-04
1430197_a_at	Pitpnm2	phosphatidylinositol transfer protein, membrane-associated 2	10.3	5.36E-05
1428942_at	Mt2	metallothionein 2	9.7	5.12E-06
1418808_at	Rdh5	retinol dehydrogenase 5	9.7	9.82E-05
1427221_at	MGI:2143217	X transporter protein 3 similar 1 gene	9.3	1.29E-04
1430817_at	Samd7	sterile alpha motif domain containing 7	8.6	2.66E-04
1447494_at	D7Bwg0826e	DNA segment, Chr 7, Brigham & Women's Genetics 0826 expressed	7.2	5.77E-06
1428352_at	Arrdc2	arrestin domain containing 2	7.1	6.05E-05
1446587_at	-	Transcribed locus	6.9	2.32E-04
1424838_at	A330049M08Ri	RIKEN cDNA A330049M08 gene	6.8	1.21E-04
1426117_a_at	Slc19a2	solute carrier family 19 (thiamine transporter), member 2	6.8	1.80E-04
1430357_at	H3f3b	H3 histone, family 3B	6.3	5.84E-05
1422557_s_at	Mt1	metallothionein 1	6.3	5.42E-06
1454991_at	Slc7a1	solute carrier family 7 (cationic amino acid transporter, y+ system), member 1	6.1	1.39E-04
1429348_at	Sema3c	semaphorin 3C	5.9	3.55E-04
1442366_at	6820408C15Ri	RIKEN cDNA 6820408C15 gene (6820408C15Rik), mRNA	5.6	6.15E-05
1441673_at	C80120	expressed sequence C80120	5.3	1.39E-04

DOWNREGULATED				

1429169_at	Rbm3	RNA binding motif protein 3	19.41	2.54E-04
1435692_at	LOC622320	similar to retinoic acid, EGF, and NGF upregulated	7.02	3.79E-05
1447363_s_at	Bub1b	budding uninhibited by benzimidazoles 1 homolog, beta (S. cerevisiae)	6.36	8.09E-06
1416961_at	Bub1b	budding uninhibited by benzimidazoles 1 homolog, beta (S. cerevisiae)	5.41	1.20E-06
1444172_at	-	Transcribed locus	4.72	2.64E-04
1435158_at	Rbm12b	RNA binding motif protein 12B	4.40	6.09E-05
1425083_at	Otor	otoraplin	4.03	2.21E-04
1445709_at	Mdm1	transformed mouse 3T3 cell double minute 1	3.97	1.64E-04
1450953_at	Wdr39A	WD repeat domain 39	3.70	1.93E-04
1456723_at	Prr14	Proline rich 14	3.49	2.35E-05
1456834_at	Ibrdc2	IBR domain containing 2 (Ibrdc2), mRNA	3.45	1.76E-04
1442051_at	His2h3cl	histone 2, H3c1	3.39	2.63E-04
1437647_at	Dido1	death inducer-obliterator 1	3.38	6.54E-05
1421379_at	Zfp354b	zinc finger protein 354B	3.28	3.41E-04
1424852_at	Mef2c	myocyte enhancer factor 2C	3.22	1.40E-04
1429655_at	Nudcd1	Nudcd1 NudC domain containing 1 3.19 3.84E-04	3.19	3.84E-04
1442249_at	-	Transcribed locus	3.11	1.03E-04
1416920_at	Rbm4	RNA binding motif protein 4	3.11	1.44E-04
1460107_at	1700129I04Rik	RIKEN cDNA 1700129I04 gene	3.11	1.74E-04
1453010_at	Iws1	IWS1 homolog (S. cerevisiae)	2.98	2.80E-04

FC: fold change; pVal: p-value

Most interestingly, expression of several genes with a potential capacity to protect photoreceptors against light-induced cell death was upregulated (Table 1). To this group belong *p21 *and *Bcl2l10*. In addition, genes belonging to oxidative stress response pathways or lipid metabolism like metallothioneins (*Mt1 *and *Mt2*), transthyretin (*Ttr*) and paraoxonase1 (*Pon1*) were induced as was the expression of adrenomedullin (*Adm*) which was previously shown to respond to hypoxic conditions [26] and to have neuroprotective properties [27].

Some pro-apoptotic genes were downregulated after hypoxic preconditioning (Table 1) like *Mef2c*, a transcription factor involved in neuronal loss in Parkinson's disease [28] and in the regulation of apoptosis in macrophages [29]. The genes belonging to the Rbm family of genes are also of high interest since some members of this family are known to have an impact on apoptosis regulation [30].

### Verification of Affymetrix microarray data

**Figure 3:**
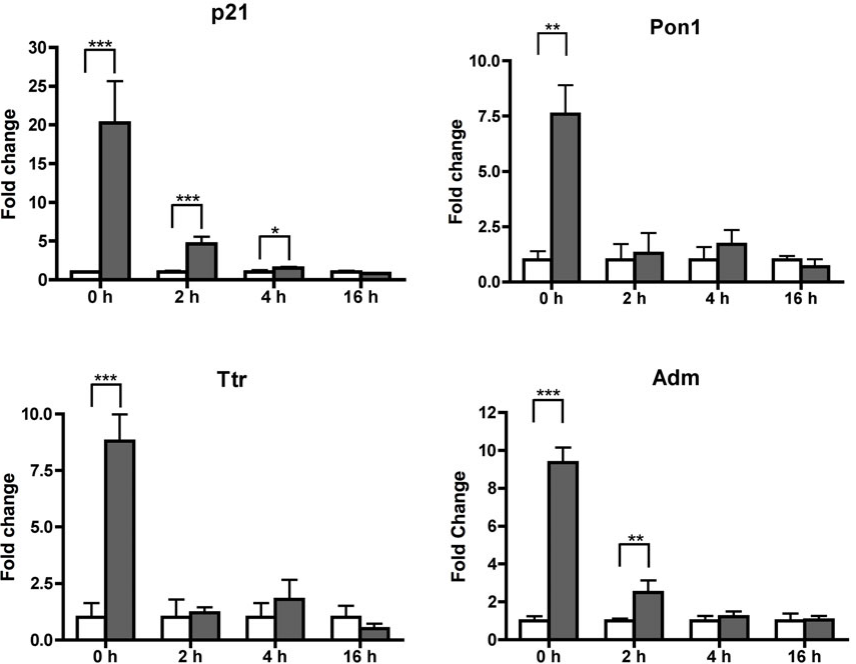
**Expression of candidate genes analyzed by real-time PCR**. Expression profile of *p21 *(*Cdkn1a*), *Pon1 *(paraoxonase 1) *Ttr *(transthyretin) and *Adm *(adrenomedullin) immediately after hypoxic exposure (0 h), or after a period of 2 h, 4 h or 16 h of reoxygenation as determined by real-time PCR. Fold-changes are expressed relative to normoxic controls of respective time points, which were set to one. n = 5 for each treatment and time point. White bars: samples of normoxic retinas, grey bars: samples of hypoxic preconditioned retinas; *** p ≥ 0.001, ** p ≥ 0.01, * p ≥ 0.05).

The expression of a total of 32 genes identified by the microarrays was tested by realtime PCR. Differential expression in response to hypoxia was verified for 21 (66%) genes (Fig. 3 and additional file 3). Quality control analysis showed that the integrities of both retinal RNA and chip surfaces were high [QC analysis, see additional file 1] and are thus unlikely to be responsible for the somewhat low confirmation frequency.

Since many of the genes which were not confirmed by real-time PCR to be differentially regulated had a low fold-change on the microarrays, we used only genes with a minimal fold change of 2 for the investigations of functionally related groups (see below). Figure 3 shows the real-time PCR data of 4 selected genes with potential neuroprotective properties (*p21*, *Pon1*, *Ttr*, *Adm*). Consistent with the microarray results, these genes were strongly upregulated immediately after hypoxia and returned quickly to normal expression levels during reoxygenation.

### Biological functional groups and pathway analysis

**Figure 4:**
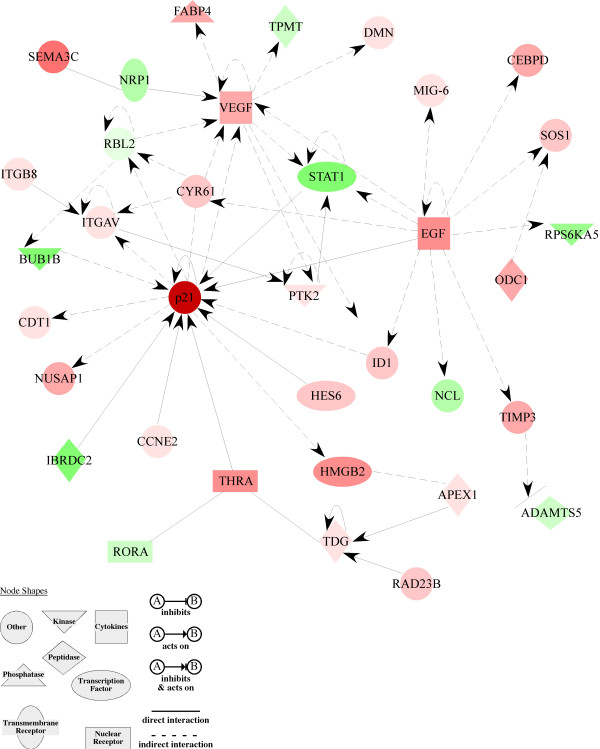
**The most prominently affected gene network discovered by Ingenuity Pathway Analysis**. Network was classified as: *Cellular growth & proliferation, DNA replication, recombination & repair*. Pathway contains pro survival genes like *Egf*, *Vegf *and *p21*, which occupy central positions in this network. Red: induction; green: repression; white: unaffected; colour intensity correlates with fold change.

The bioinformatics resource DAVID was used to study the impact of differentially expressed genes on known biological processes [31]. We obtained 39 functional groups for upregulated genes and 8 groups for downregulated genes immediately after hypoxia (H0). Significant functional groups included 'apoptosis', 'cell cycle' or 'negative regulation of transcription' (note that some genes appear in more than one group) were found in the list of upregulated genes (Table 2). Lists of all functional related groups can be found in additional file 4. Gene signalling networks affected by hypoxic preconditioning were identified using Ingenuity Pathway analysis and the complete lists of regulated genes (Fig 4 and see additional file 5). The centre of the strongest affected pathway was occupied by *p21*, one of the most strongly upregulated genes by hypoxia (Fig. 4). All genes belonging to this pathway were differentially regulated. 70% of the genes were induced indicating that the pathway was activated rather than repressed. All genes of this pathway, which showed at least a two-fold differential regulation on the chip, were tested by real-time PCR. Ten of the 16 tested genes were confirmed to be regulated by hypoxia (Table 3). This suggested that the p21-pathway was indeed strongly affected by the hypoxic preconditioning protocol. Since it is known that p21 not only inhibits cell cycle but can also repress apoptosis [32], it was considered as a strong candidate for the involvement in neuroprotection by hypoxic preconditioning.

**Table 2:** Differentially regulated genes with possible impact on cell survival and neuroprotection; detected and functionally clustered by DAVID.

**Affymetrix ID**	**Gene Symbol**	**Gene Name**	**FC**
Up-regulated			
apoptosis (p ≥ 0.032)			
1424638_at	Cdkn1a	cyclin-dependent kinase inhibitor 1a (p21)	30.5
1416434_at	Bcl2l10	Bcl2-like 10	17.0
1442025_a_at	Zbtb16	zinc finger and btb domain containing 16	3.6
1454109_a_at	Ptdsr	phosphatidylserine receptor	2.4
1420909_at, 1451959_a_at	Vegfa	vascular endothelial growth factor a	2.3
1453851_a_at	Gadd45g	growth arrest and dna-damage-inducible 45 gamma	2.3
1452050_at	Camk1d	calcium/calmodulin-dependent protein kinase id	2.1
1454903_at	Ngfr	nerve growth factor receptor (TNFR superfamily, member 16)	2.0
cell cycle (p ≥ 0.013)			
1424638_at	Cdkn1a	cyclin-dependent kinase inhibitor 1A (P21)	30.5
1416309_at	Nusap1	nucleolar and spindle associated protein 1	4.4
1454018_at	Tlk2	tousled-like kinase 2 (Arabidopsis)	4.3
1449007_at	Btg3	B-cell translocation gene 3	4.2
1424143_a_at, 1424144_at	Cdt1	retroviral integration site 2	3.4
1419024_at, 1455002_at	Ptp4a1	protein tyrosine phosphatase 4a1	2.7
1453851_a_at	Gadd45g	growth arrest and DNA-damage-inducible 45 gamma	2.3
1420909_at, 1451959_a_at	Vegfa	vascular endothelial growth factor A	2.3
1459978_x_at	-	gene model 877 (NCBI)	2.0
1435870_at	Sycp3	synaptonemal complex protein 3	2.0
negative regulation of transcription (p ≥ 0.031)			
1442025_a_at	Zbtb16	zinc finger and btb domain containing 16	3.6
1425809_at	Fabp4	Fatty acid binding protein 4, adipocyte (Fabp4), mRNA	3.5
1425895_a_at	Id1	inhibitor of DNA binding 1	3.1
1442397_at	Nfx1	nuclear transcription factor, X-box binding 1	2.7
1425732_a_at	Mxi1	Max interacting protein 1	2.1
Down-regulated			
regulation of transcription (p ≥ 0.09)			
1440343_at	Rps6ka5	ribosomal protein S6 kinase, polypeptide 5	0.42
1450034_at	Stat1	signal transducer and activator of transcription 1	0.47
1416826_a_at	Trfp	Trf (TATA binding protein-related factor)-proximal protein homolog (Drosophila)	0.41
1450953_at	Wdr39	WD repeat domain 39	0.27
1421379_at	Zfp354b	zinc finger protein 354B	0.31
1424852_at	Mef2c	myocyte enhancer factor 2C	0.31
1437647_at	Dido1	death inducer-obliterator 1	0.30
1443952_at	Thra	thyroid hormone receptor alpha	0.48
1428760_at	Snapc3	small nuclear RNA activating complex, polypeptide 3	0.40
1456723_at	Prr14	Proline rich 14	0.29

Analysis of the gene expression pattern was immediately after hypoxia; FC: fold change

**Table 3:** Real-Time PCR results of 16 genes belonging to the p21 pathway compared to the fold change (FC) detected by Affymetrix microarrays.

**Gene symbol**	**FC Affymetrix Chip**	**FC PCR**
Cdkn1a (p21)	30.5	20.3*
Sema3c	5.9	3.9*
Egf	4.7	2.8*
HMGB2	4.1	3.0*
Fabp4	3.5	5.3*
CEBPd	3.5	3.5*
Timp3	3.3	3.7*
ID1	3.1	1.8*
Rad23b	2.6	1.2
Vegf	2.3	2.0*
Stom	2.0	1.3
Hes6	1.9	1.6*
SOS1	1.9	0.9
Stat1	0.5	1.1
Thra	0.5	0.8
IBRDC2	0.3	0.9

Analysis of gene expression was immediately after hypoxia

FC: fold change. * p < 0.05

### The influence of p21 on retinal neuroprotection in the model of light induced degeneration

**Figure 5:**
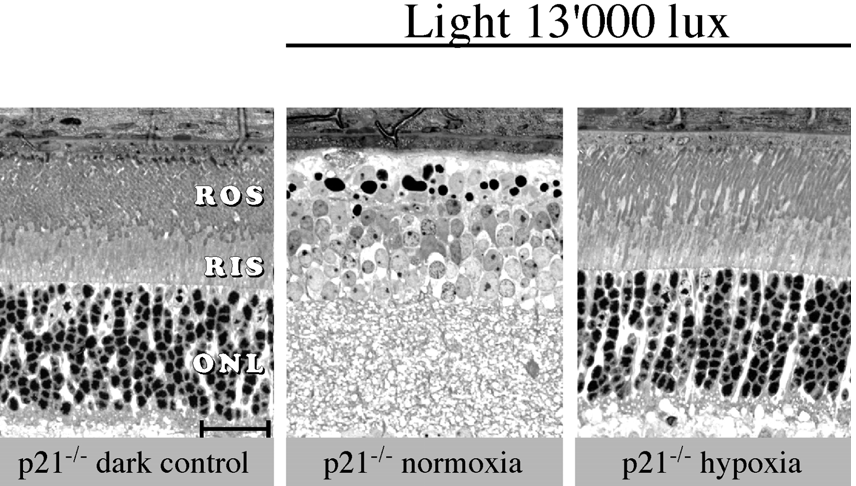
**Role of p21 in hypoxic preconditioning**. Hypoxic preconditioned (hypoxia) or normoxic (normoxia) p21^-/- ^mice were exposed to 13'000 lux of white light for 2 hours and retinal morphology was analyzed 10 days thereafter. Dark-adapted knockout mice served as controls (dark control). Hypoxic preconditioning almost completely protected the retina of p21^-/- ^mice leading to a retinal morphology indistinguishable from controls. Exposure of normoxic mice led to a complete degeneration of photoreceptors in the central retina. Shown are representative samples of control and light exposed mice. n = 2 (controls); n = 4 (light exposed mice). Abbreviations as in Fig. 1. Scale bar: 25 μm.

The hypothesis that p21 is important for neuroprotection in the retina after hypoxic preconditioning was directly tested using p21 knockout animals. Both, normoxic and hypoxic preconditioned p21^-/- ^mice were exposed to high intensity visible light for 2 hours and retinal morphology was analyzed 10 days thereafter (Fig. 5). As expected, normoxic control p21^-/- ^mice showed strong damage after light exposure with the loss of all photoreceptors in the central retina. If p21 was involved in neuroprotection after hypoxic exposure, preconditioned p21^-/- ^mice should show an increased susceptibility to light damage as compared to wild type mice. However, photoreceptors of the p21 knockout mice were completely protected after preconditioning (Fig. 5). The quantification of cell death by biochemical assays (data not shown) supported our conclusion that p21 does not contribute significantly to the neuroprotective effect observed after hypoxic preconditioning. Furthermore, most genes identified by Ingenuity Pathway analysis as being part of the p21 signalling network were similarly regulated in the presence or absence of functional p21. The only exception was Semaphorin 3c (*Sema3c*), which showed no hypoxic upregulation in the absence of p21 (data not shown).

## Discussion

Hypoxic preconditioning is strongly neuroprotective and prevents photoreceptor apoptosis after exposure to high levels of visible light [9]. The transcription factors HIF-1 and Stat3 are activated. This suggests differential regulation of the expression of various target genes which was confirmed by the detection of 431 differentially regulated genes immediately after hypoxia. More than 50% of these genes showed at least a 2-fold difference in the expression level as compared to normoxic retinas. Among those were also *Rgr *and *Lrat*, two genes highly expressed in the retinal pigment epithelium. Genes normally not or only barely expressed in the neuronal retina may easily reach a high-fold induction when the tissue is contaminated with neighbouring cells expressing the respective gene at high levels. Low levels of oxygen during hypoxic preconditioning may have altered the physical interaction properties between neuronal retina and retinal pigment epithelium (RPE) leading to an increased contamination rate of the retina by cells of the RPE during tissue isolation. Thus, fold inductions have to be interpreted cautiously.

Reoxygenation caused the rapid return to a normal gene expression pattern. This is in line with a model of hypoxic preconditioning in brain where it was shown that differential gene regulation was low between 12 and 18 hours of reoxygenation [33]. In models of ischemic preconditioning (IPC), however, differential gene expression is observed immediately after the stimulus until up to 7 days after preconditioning [34-36]. This goes together with a long lasting neuroprotective effect of IPC observed in the retina [10] and in brain [37] suggesting that mechanisms of IPC may differ from those of acute hypoxic preconditioning. An extended neuroprotection by hypoxic preconditioning may be achieved by the repetitive exposure to low oxygen [12].

### Similarities of hypoxic preconditioning and IPC

Although mechanisms of hypoxic preconditioning and IPC may differ, few genes were found to be differentially regulated in both types of preconditioning protocol. Among those are metallothionein 2 (Mt2), C/ebpd (an apoptosis-related transcription factor) and p21 [35,38]. The identification of these genes makes them strong candidates for playing a role in general retinal neuroprotection.

An involvement of p21 was directly tested using the respective knockout animal. As a HIF-1 target gene [39], p21 was not only very strongly regulated but was also at the center of a highly regulated gene network (Fig. 4). Although p21 can be pro-apoptotic [40] and can trigger non-apoptotic cell death [41], it is also known to have antiapoptotic properties [42]. However, the test of p21 knockout mice in the model of light induced degeneration revealed no significant impact of *p21 *on neuroprotection against light damage. Despite the lack of *p21*, all other genes of the p21 pathway (except for *Sema3c*) showed the same response to hypoxic preconditioning as in wild type mice. This raises the possibility that other genes of the p21 network might influence retinal neuroprotection. Specific candidates are *Timp3 *which has been reported to be a promoter of apoptosis through the inhibition of metalloproteinases [43] and *Egf *which has proven anti-apoptotic properties [44].

*Mt2*, as a gene also detected in both preconditioning schemes, may play an important role as a scavenger of free radicals [45]. It is interesting to note that metallothioneins are also induced after ischemic preconditioning of the rat spinal cord [34] and that they have been reported to be neuro- and cardioprotective, respectively, in various degenerative models [46,47]. In addition, metallothioneins have been shown to be induced in light-damaged retinas [48] and to protect retinas from oxidative stress caused by the glutamate analogue NMDA [49]. Further experiments are clearly needed to evaluate the impact of this protein in retinal neuroprotection.

The low similarity of the transcriptome after IPC and hypoxic preconditioning may be surprising but might be based on the different nature of the preconditioning protocols. Whereas IPC normally uses a very short (minutes) ischemic stimulus followed by reperfusion, our protocol of hypoxic preconditioning uses exposure to 6 hours of low oxygen concentrations followed by the immediate analysis. The different length of exposure to low oxygen and the interrupted supply of nutrients in one (IPC) but not the other protocol might essentially explain the differences in the gene expression patterns.

### Strong candidate genes for neuroprotection: *Adm*, *Pon1*

Adrenomedullin (*Adm*) is a multifunctional protein involved in angiogenesis, cancer promotion, host defence and neuroprotection [50]. Elevated levels of Adm were found in plasma of patients suffering from retinitis pigmentosa [51]. Previous reports identified *Adm *as a target gene of HIF-1α [52,53] linking it to a possible HIF – mediated protection mechanism.

Interestingly, some genes which so far were not described in the context of hypoxia, like paraoxonase 1 (*Pon1*), were also highly induced. Pon1 is a high-density lipoprotein (HDL) associated enzyme which plays a major role in the prevention of lipid peroxidation [54,55]. Since retinal degeneration involves oxidative stress and inhibition of lipid peroxidation protects against light damage [56]* Pon1 *may have an important role in retinal protection after hypoxic preconditioning. Recently, *Pon1 *levels were found to be reduced in serum of AMD patients whereas a marker for oxidative stress was elevated [57]. This may suggest that elevated levels of *Pon1 *in our model might reduce oxidative stress and prevent photoreceptor degeneration. Interestingly, C57Bl/6 mice which have a reduced sensitivity to light damage show a higher basal expression of *Pon1 *than light sensitive strains (data not shown). If the anti-oxidative enzyme Paraoxonase 1 was involved in the protection of the retina against oxidative damage, the different basal expression levels of *Pon1 *might contribute to the different light damage susceptibilities of various mouse strains.

### Additional genes with potential neuroprotective function

Bcl2-like 10 (*Bcl2l10*) is a anti-apoptotic member of the Bcl2 family [58] acting to suppress cell death by preventing cytochrome c release, casp-3 activation and mitochondrial membrane collapse [59]. However, retinal degeneration induced by acute light exposure may not depend on cytochrome c release or caspase activation [60]. Therefore, upregulation of Bcl2l10 might not be responsible for photoreceptor protection by hypoxic preconditioning.

Induction of the HIF-1 target gene *Vegfa *is an attempt to increase tissue oxygen levels by improving blood circulation through the formation of new vessels [61]. In the retina Vegfa is also recognized as a pro-survival factor protecting retinal neurons against ischemic injury [62]. However, Vegfa is discussed to have also pro-apoptotic properties [63] and its potential role in the preconditioning scheme is unclear. *Ptdsr *encodes a posphatidylserine receptor involved in the clearance of apoptotic cells [64] and it has been shown that lack of Ptdsr activity can increase tissue damage through the stimulation of apoptosis in cells neighbouring apoptotic cells [65]. *Ptdsr *is also involved in the elimination of apoptotic debris of dying photoreceptors by macrophage-mediated phagocytosis which is important for the maintenance of retinal tissue integrity [66].

Downregulated genes with a possible impact on cell death included *Mef2c *and genes of the Rbm family of protein. *Mef2c *triggers apoptosis in macrophages [29] and may be involved in dopaminergic neuron death in Parkinson's disease [28]. Because macrophages seem to play an important role in light induced apoptosis [67,68] a potential influence on neuroprotection may be possible but needs further investigation. This is also true for the identified members of the Rbm family. Although these proteins have been implicated in the modulation of apoptosis [30], and downregulation of *Rbm3 *has been specifically connected to the regulation of cell cycle progression [69] and the inhibition of apoptosis [70], their role is still controversial.

## Conclusion

Since hypoxia can either lead to adaptation and protection [71] or to apoptosis [72] it may not be surprising that we identified several genes which may rather be involved in promoting apoptosis than in its inhibition. Neuroprotection by hypoxic preconditioning may thus depend on a balance between numerous anti- and proapoptotic factors. The loss of individual proteins like p21 may not be sufficient to shift the balance towards apoptosis. Likewise, it might require several different antiapoptotic factors to fully protect the retina. Full neuroprotection may only be achieved by controlling the central regulators of the hypoxic response like the transcription factors HIF and/or STAT3.

## Methods

### Animals, hypoxic preconditioning and light damage

Animals were treated in accordance with the regulations of the Veterinary Authority of Zurich and with the statement of 'The Association for Research in Vision and Ophthalmology' for the use of animals in research. BALB/c mice were purchased from Harlan (The Netherlands) and p21^-/- ^mice on a mixed Bl/6;129S2 background were obtained from Jackson Laboratory (Bar Harbor, USA). All mice were homozygous for the light sensitive Rpe65_450Leu _variant [73]. Hypoxic preconditioning (6% O_2 _for 6 hours) was performed as described previously [9]. Reoxygenation was allowed in darkness for 4 h, 8 h, 12 h and 16 h in normal room air. After reoxygenation BALB/c mice were exposed to 5'000 lux of white fluorescent light for 1 h and analyzed at time points as indicated.

Pupils of p21^-/- ^animals (pigmented) were dilated in dim red light using 1% Cyclogyl (Alcon, Cham, Switzerland) and 5% Phenylephrine (Ciba Vision, Niederwangen, Switzerland) 45 minutes prior to illumination. Light dose (13'000 lux) and exposure duration (2 h) was adjusted according to the decreased light damage susceptibility of this mouse strain. After light exposure animals remained in darkness until analyzed or at the most for 36 h.

For morphology mice were sacrificed 36 h or 10 days after light exposure and eyes were enucleated and processed as previously described [74].

### RNA isolation and Affymetrix microarrays

Retinas were isolated immediately, 2 h, 4 h and 16 h after hypoxic preconditioning, frozen in liquid nitrogen and stored at -70°C. Normoxic controls were treated in parallel and collected at the same time points. For Affymetrix microarrays 3 retinas of 3 different mice were pooled. This procedure was repeated 3 times to generate independent biological triplicates. RNA was extracted using the RNeasy isolation kit (Qiagen, Hilden, Germany), including a DNase treatment to digest residual genomic DNA. RNA was processed according to standard procedures and hybridized to Affymetrix GeneChip^® ^Mouse Genome 430 2.0 microarrays. The 3 experimental replicates were hybridized independently resulting in three microarray replicates per condition. In total, 24 Affymetrix gene chips were hybridized with RNA from 72 retinas of 72 mice.

### Quality control (QC) and Affymetrix microarray analysis

To analyze the quality of the results after gene chip hybridization we employed RReporterGenerator [75] combining Affymetrix-style QC, RMA and residual QC. The complete report is available in additional file 1.

Affymetrix raw gene expression data were summarized and normalized using the GCRMA procedure [76]. The data were filtered in order to remove probe-sets with constant low-level expression. Probe-sets were removed which showed replicate means for a given time-point for both treatments below the threshold separating the two peaks of the bimodal distribution of signal-intensity values. This procedure was performed independently for each of the differential testing procedures. The filtered data-sets were subsequently subjected to t-tests with multiple testing correction and control of the false discovery rate (FDR) using OCplus package [77] available under Bioconductor [78]. By comparing the plotted number of differentially expressed genes at various FDR levels, corresponding threshold values for the maximum acceptable FDR were chosen with the aim of keeping homogenous groups with similar FDR together.

To group differentially regulated genes according to their biological function the Affymetrix IDs were imported into the Database for Annotation, Visualization and Integrated Discovery (DAVID) from the National Institute of Allergy and Infectious Diseases (NIAID), NIH [31,79] and into Ingenuity Pathway Analyses from Ingenuity Systems [80].

### Real-time PCR

cDNA was prepared from equal amounts of total retinal RNA, using oligo(dT) primers and M-MLV reverse transcriptase (Promega, Madison, WI, USA). 10 ng of cDNA was amplified in a LightCycler 480 instrument (Roche Diagnostics AG, Rotkreuz, Switzerland) using LightCycler 480 SYBR Green I Master Mix (Roche Diagnostics AG) and appropriate primer pairs [see additional file 6]. mRNA levels were normalized to β-actin and relative values were calculated using a respective calibrator.

### Western blotting

Retinas were homogenized in 0.1 M Tris/HCl (pH 8.0) by sonification at 4°C. The protein content was determined using a Bradford assay (Bio-Rad, Munich, Germany). Protein extracts were mixed with SDS sample buffer and incubated for 10 min at 90°C. Proteins were separated by SDS-PAGE and blotted onto a nitrocellulose membrane. After blocking with 5% non-fat dry milk (Bio-Rad, Munich, Germany) in TBST (Tris/HCl 10 mM, pH 8; 150 mM NaCl; 0.05% Tween-20) membranes were incubated with primary antibodies at 4°C over night. Primary antibodies used were: rabbit anti-HIF-1α (Novus Biologicals NB 100–479; 1:1000), rabbit anti-phospho-STAT3 (Cell Signalling; 1:500), rabbit anti-STAT3 (Cell Signalling 1:1000) and goat anti-β-actin (Santa Cruz; 1:1000). After incubation with horseradish peroxidase labelled secondary antibodies for 1 h at room temperature the protein bands were visualized by the application of a chemiluminescent substrate (PerkinElmer, Boston, USA) and exposure to a Super RX film (Fujifilm, Dielsdorf, Switzerland).

## Authors' contributions

MT, RF, MS and CG performed experiments. MT, WR and OP analyzed microarray data. Study was designed by MT, RF, AW and CG. Manuscript was written by MT and CG.

## Supplementary Material

Additional file 1 **Fig 1 QC Analyses**. Results of QC analyses using RReporterGenerator. File contains report of QC analyses performed by RReporterGenerator. Click here for file

Additional file 2 **Table 1, 2, 3 Gene Lists of differentially regulated genes**. Table 1 List of differentially regulated genes immediately after hypoxia (H0). Table 2 List of differentially regulated genes 2 h after hypoxia (H2). Table 3 List of differentially regulated genes 4 h after hypoxia (H4). File contains all genes found to be differentially regulated immediately after hypoxia (Table 5 H0), 2 h after hypoxia (Table 6, H2) and 4 h after hypoxia (Table 7, H4). Click here for file

Additional file 3 **Table 4 Real time PCR results**. Table 4 Real-Time PCR results of the expression of genes detected by Affymetrix microarrays as differentially regulated by hypoxic preconditioning. File contains all genes revealed by microarray analyses, which were tested by real time PCR. Click here for file

Additional file 4 **Table 5,6 List of genes functionally clustered by DAVID**. Table 5 Differentially up-regulated genes functionally clustered by DAVID. Table 6 Differentially down-regulated genes functionally clustered by DAVID. File contains functional clusters of genes found to be differentially regulated immediately after hypoxia (H0 data). Click here for file

Additional file 5 **Fig 2, 3 Hypoxic preconditioning gene networks discovered by Ingenuity Pathway analysis**. Fig 2 affected gene network discovered by Ingenuity Pathway Analysis and classified as: DNA replication, recombination, cell cycle and cancer. Fig. 3 affected gene network discovered by Ingenuity Pathway Analysis and classified as: Cell death, cellular development, hematological system development & function. File contains schemes of pathways, which are affected by hypoxic preconditioning. Some genes connected to cMyc (Fig. 7) and to TGF-b (Fig. 8) are differentially regulated. Click here for file

Additional file 6 **Table 7 Primer Sequences and product sizes**. Table 7 Primer pairs used for real time PCR. File contains sequences of Primers used for real time PCR and the expected/obtained product sizes in bp. Click here for file
