# Supplementary material for: Analysis of the retinal gene expression profile after hypoxic preconditioning identifies candidate genes for neuroprotection
Source: BMC Genomics. 2008 Feb 8;9:73. doi: 10.1186/1471-2164-9-73 (PMC2270833; doi:10.1186/1471-2164-9-73)
Supplement: Additional file 1 — Fig 1 QC Analyses. Results of QC analyses using RReporterGenerator. File contains report of QC analyses performed by RReporterGenerator. [file 1471-2164-9-73-S1.pdf]

Affymetrix Batch QC using RReportGenerator and R

September 21, 2007

This document was generated by Analysis Type File *automAffyQC2.Rnw'*, Version: 1.0.8
a protocol for automated QC analysis for Affymetrix expression array data.
Written by wolfgang.raffelsberger@igbmc.u-strasbg.fr, LGBI, IGBMC (Strasbourg, France).

QC analysis of 24 cel-files of type Mouse430.2 found in :

```
[1] "/genomics/g6/CELs/retine/Gri_Hypox/all"
```

page 3
The blots address original PM values, either as boxplot or as density estimate for the signal distribution (similar to packages simpleaffy and affyQCReport).

page 4
This figure shows the QC plot from the simpleaffy package. Briefly, these plots show the 3' to 5' ratio for spiked-in and control genes (typically with triangles for b-Actin and squares fro GAPDH). The dot with the vertical line (heading to 0) shows the scaling factor. Finally, the percentage of present calls and value of average background are shown in the left part of the image.

page 5
This pair of graphs shows the RLE (top) and NUSE (bottom) plots from the affyPLM package. The RLE compares expression values on each array against median expression values, for a probeset across all arrays, and the NUSE plot shows the standard errors for each gene standardized across all arrays.

page 6
This page shows false color images for the residuals (from the affyPLM package, with red intensities corresponding to positive residuals and blue to negative residuals).

page 7
MA plots (from affyPLM package) of each array against a synthetic median array (constructed from probe-wise medians). The red line represents a lowess fit to the scatter plot and is helpful in indicating non-linear relationships.

page 8
In the top part the RNA degaradation plot shows the average intensity with respect to the sorted 5' to 3' position of the probes in the target-sequence. Depending on the type of microarray specific patters can be observed (see also Bolstad and Gentleman et al) . The lower figure shows a density estimate for the signal distribution of data resulting from RMA.

page 9
Similarity between GCRMA summarized samples measured as Eucliedean Distance.
Top: Distance matrix for all pairwise comparisons. Red cells indicate very similar samples.
Bottom: Dendrogram from Hierarchical Clustering with Bootstrap p-Values. Approximately Unbiased (AU) p values are shown in red, Bootstrap Probability (BP) in green. AU might be a better approximation to unbiased p-value than BP values (Suzuki et al).

The aim of this report is to provide information on multiple QC aspects for a set of Affymetrix arrays. The interpretation of QC parameters and QC plots should be done with care since this may lead to delicate decisions. For further information and details about the plots shown in this report please look at the references section.

The analysis was run on September 21, 2007 , using R version 2.5.1 on a x86-64-unknown-linux-gnu system.

..total computing time : 417.6 min

Overview of Arrays Used

array index	sample names
1 ...	mz_251105_mz_H0_1
2 ...	mz_251105_mz_H0_2
3 ...	mz_251105_mz_H0_3
4 ...	mz_251105_mz_H16_1
5 ...	mz_251105_mz_H16_2
6 ...	mz_251105_mz_H16_3
7 ...	mz_251105_mz_H2_1
8 ...	mz_251105_mz_H2_2
9 ...	mz_251105_mz_H2_3
10 ...	mz_251105_mz_H4_1
11 ...	mz_251105_mz_H4_2
12 ...	mz_251105_mz_H4_3
13 ...	mz_251105_mz_N0_1
14 ...	mz_251105_mz_N0_2
15 ...	mz_251105_mz_N0_3
16 ...	mz_251105_mz_N16_1
17 ...	mz_251105_mz_N16_2
18 ...	mz_251105_mz_N16_3
19 ...	mz_251105_mz_N2_1
20 ...	mz_251105_mz_N2_2
21 ...	mz_251105_mz_N2_3
22 ...	mz_251105_mz_N4_1
23 ...	mz_251105_mz_N4_2
24 ...	mz_251105_mz_N4_3

Boxplots for PM Values

data from: /genomics/g6/CELs/retine/Gri_Hypox/all

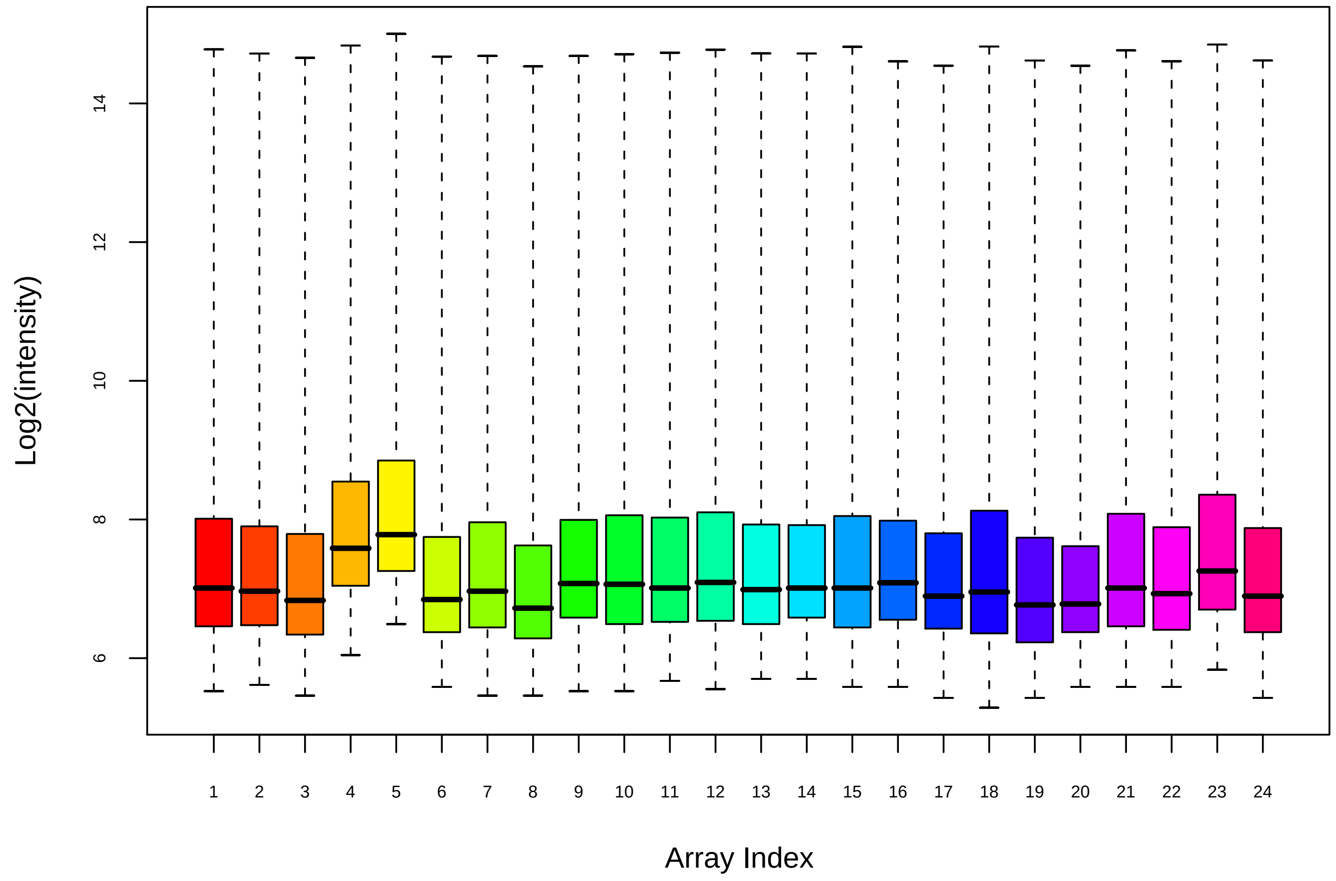

Histogram for PM Values (kernel density estimate)

data from: /genomics/g6/CELs/retine/Gri_Hypox/all

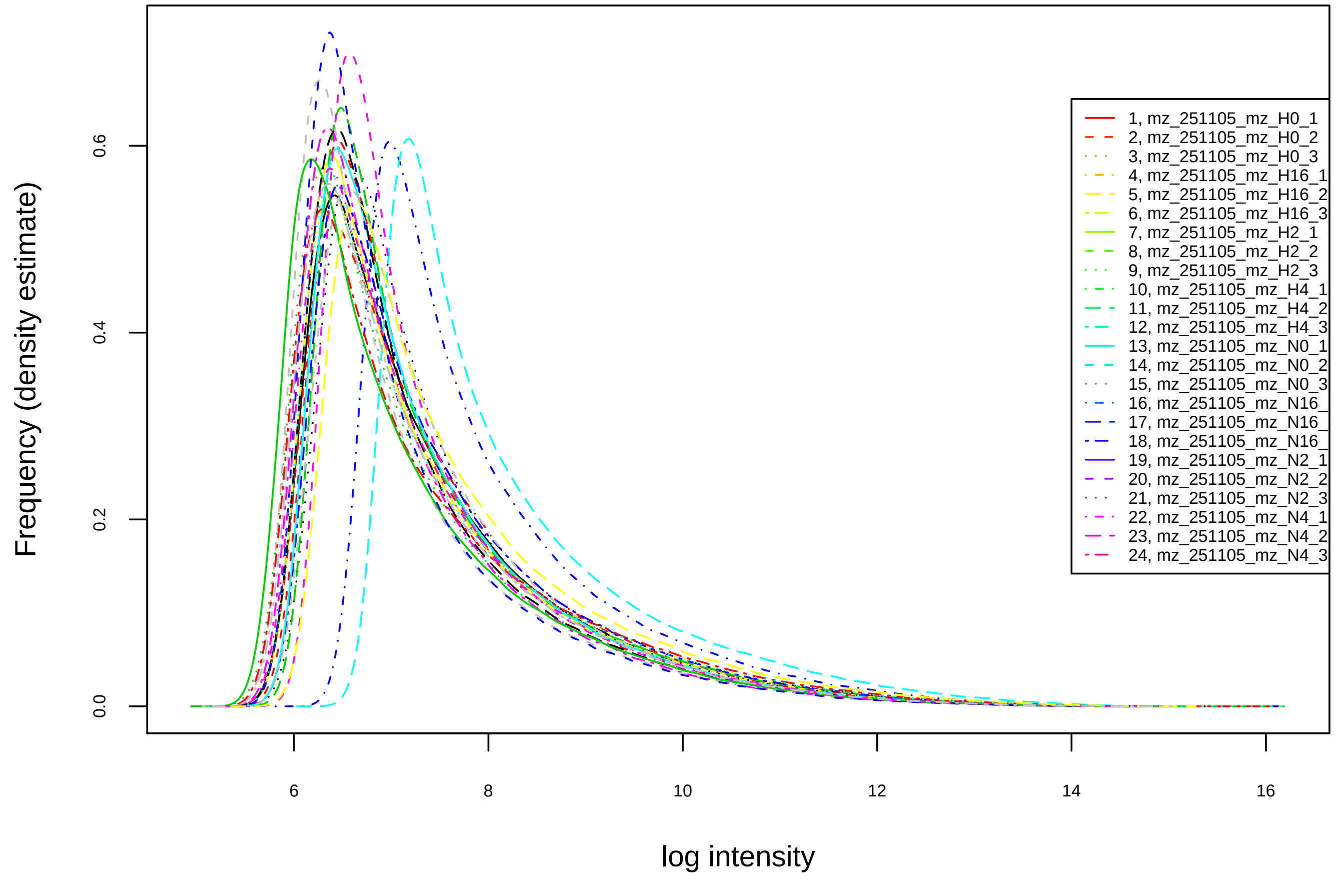

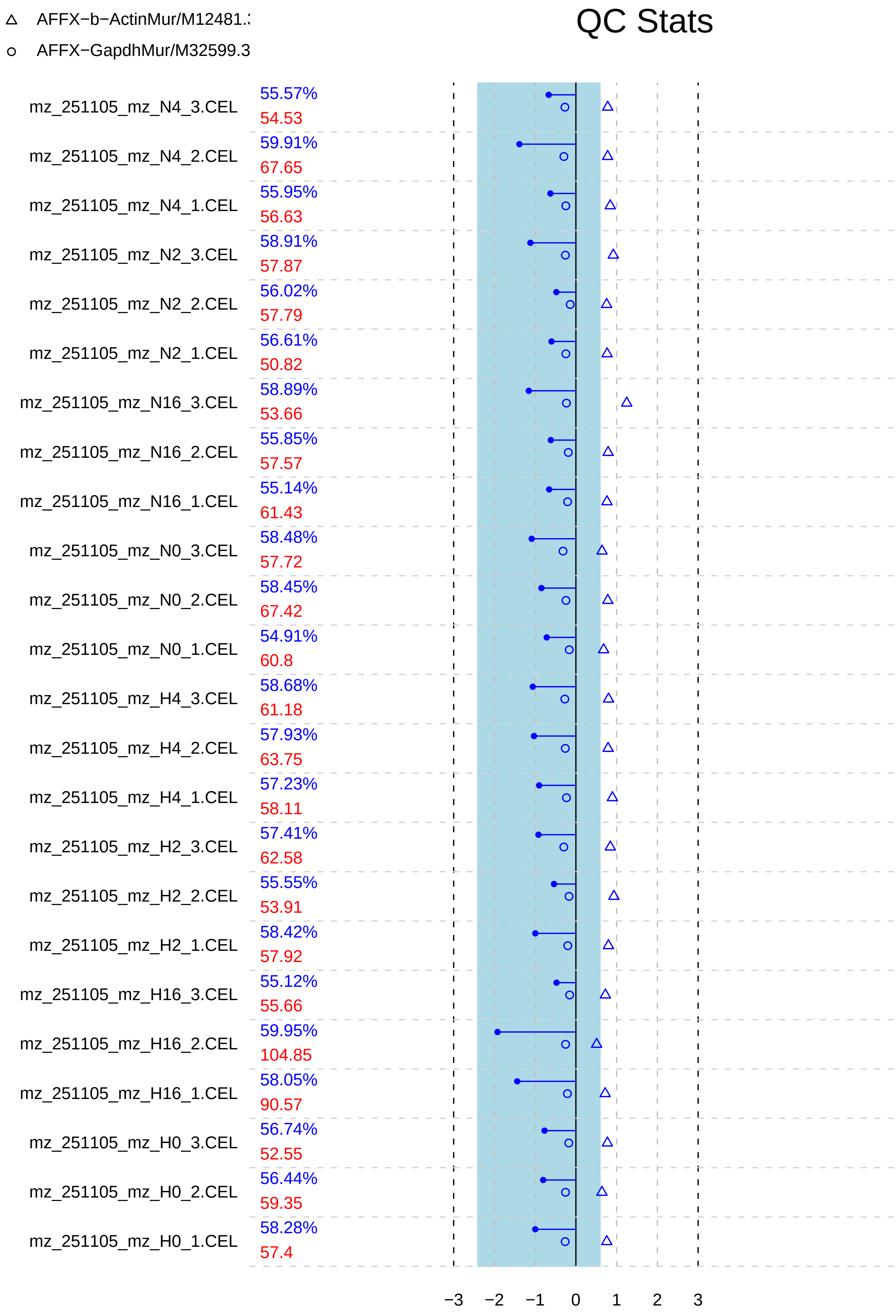

AFFX−b−ActinMur/M12481.3
AFFX−GapdhMur/M32599.3
QC Stats
mz_251105_mz_N4_3.CEL
55.57%
54.53
mz_251105_mz_N4_2.CEL
59.91%
67.65
mz_251105_mz_N4_1.CEL
55.95%
56.63
mz_251105_mz_N2_3.CEL
58.91%
57.87
mz_251105_mz_N2_2.CEL
56.02%
57.79
mz_251105_mz_N2_1.CEL
56.61%
50.82
mz_251105_mz_N16_3.CEL
58.89%
53.66
mz_251105_mz_N16_2.CEL
55.85%
57.57
mz_251105_mz_N16_1.CEL
55.14%
61.43
mz_251105_mz_N0_3.CEL
58.48%
57.72
mz_251105_mz_N0_2.CEL
58.45%
67.42
mz_251105_mz_N0_1.CEL
54.91%
60.8
mz_251105_mz_H4_3.CEL
58.68%
61.18
mz_251105_mz_H4_2.CEL
57.93%
63.75
mz_251105_mz_H4_1.CEL
57.23%
58.11
mz_251105_mz_H2_3.CEL
57.41%
62.58
mz_251105_mz_H2_2.CEL
55.55%
53.91
mz_251105_mz_H2_1.CEL
58.42%
57.92
mz_251105_mz_H16_3.CEL
55.12%
55.66
mz_251105_mz_H16_2.CEL
59.95%
104.85
mz_251105_mz_H16_1.CEL
58.05%
90.57
mz_251105_mz_H0_3.CEL
56.74%
52.55
mz_251105_mz_H0_2.CEL
56.44%
59.35
mz_251105_mz_H0_1.CEL
58.28%
57.4
−3 −2 −1 0 1 2 3

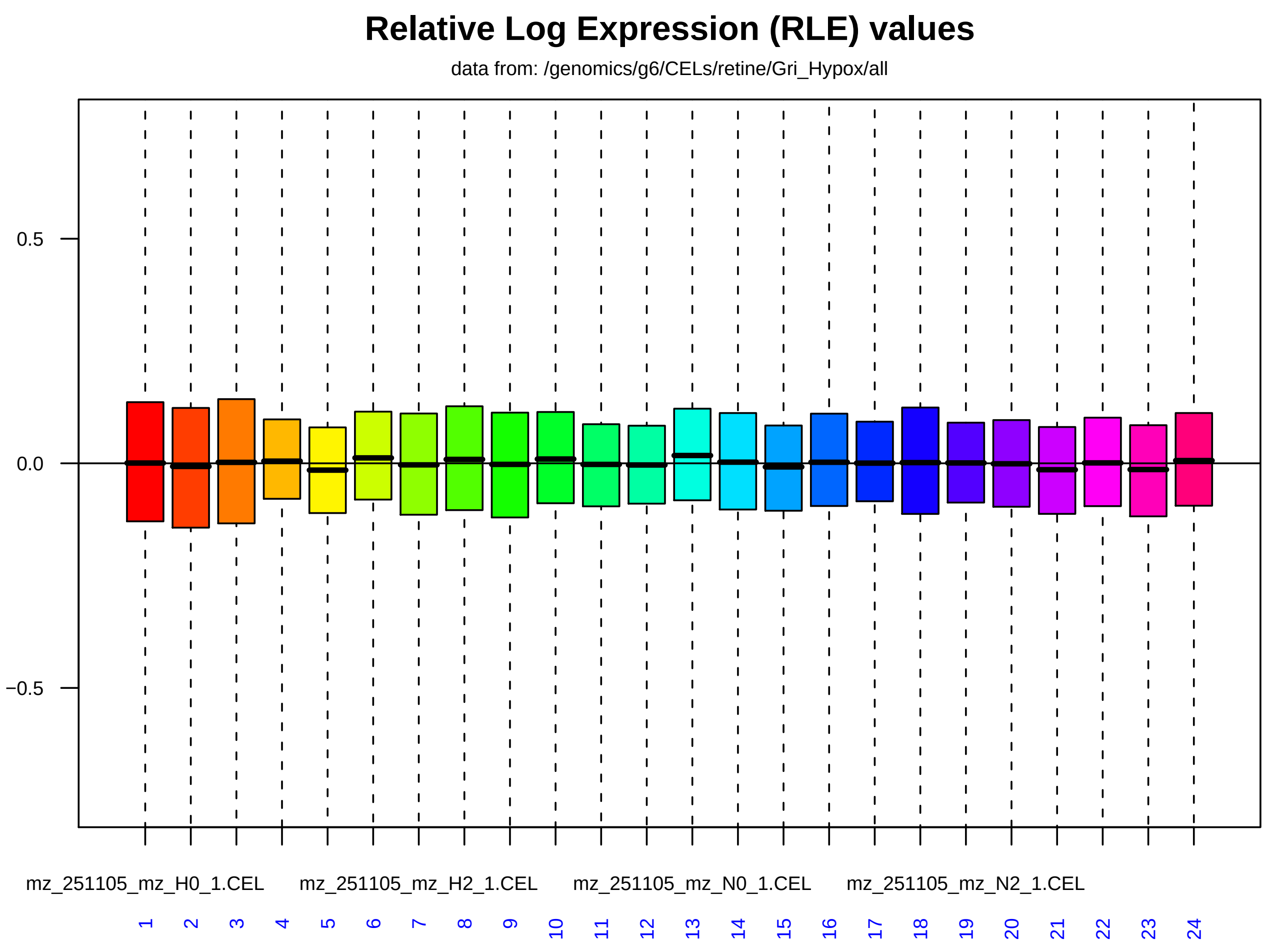
Relative Log Expression (RLE) values
data from: /genomics/g6/CELs/retine/Gri_Hypox/all
0.5
0.0
−0.5
mz_251105_mz_H0_1.CEL
mz_251105_mz_H2_1.CEL
mz_251105_mz_N0_1.CEL
mz_251105_mz_N2_1.CEL
1 2 3 4 5 6 7 8 9 10 11 12 13 14 15 16 17 18 19 20 21 22 23 24

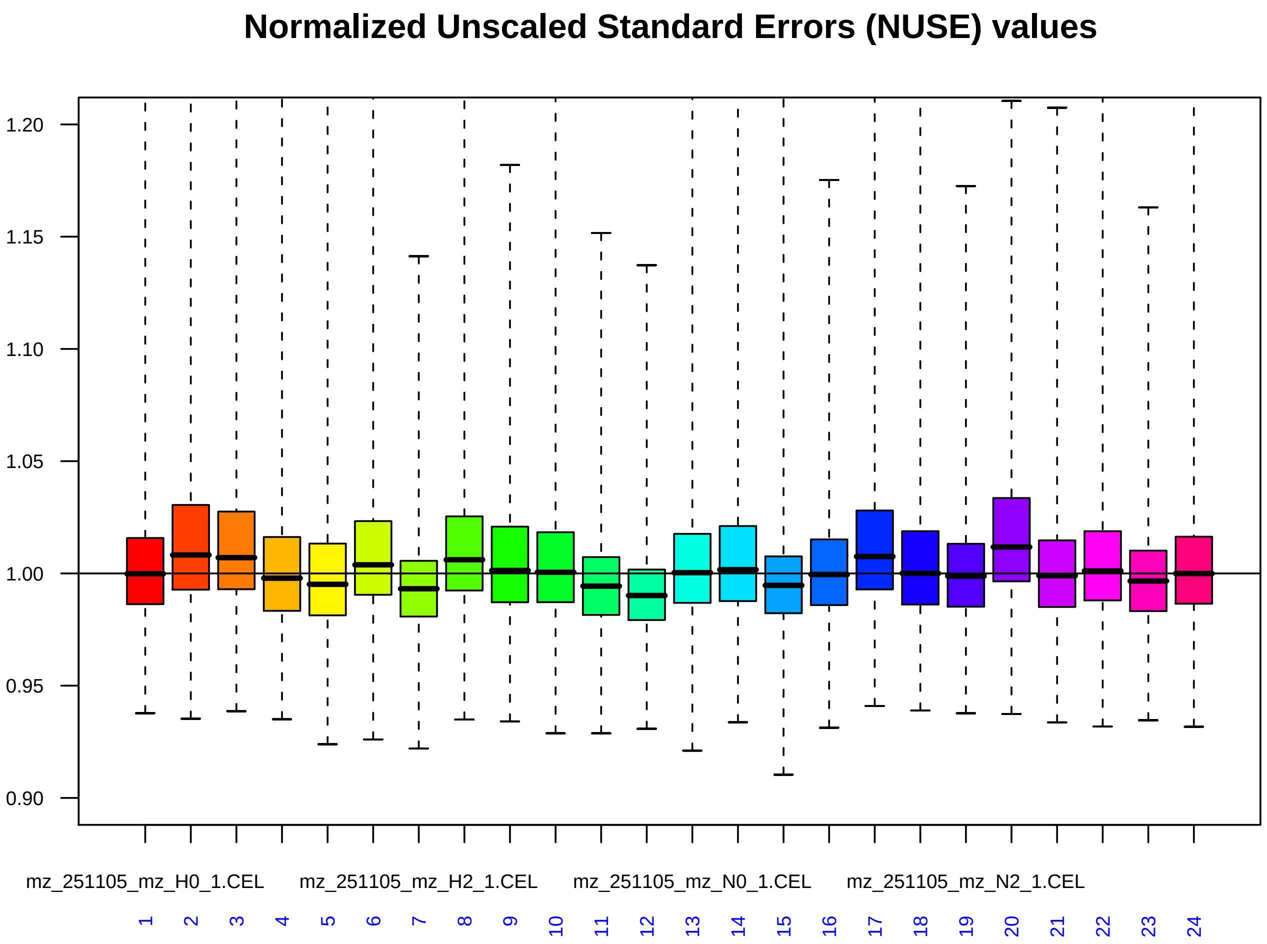
Normalized Unscaled Standard Errors (NUSE) values
1.20
1.15
1.10
1.05
1.00
0.95
0.90
mz_251105_mz_H0_1.CEL
mz_251105_mz_H2_1.CEL
mz_251105_mz_N0_1.CEL
mz_251105_mz_N2_1.CEL
1 2 3 4 5 6 7 8 9 10 11 12 13 14 15 16 17 18 19 20 21 22 23 24

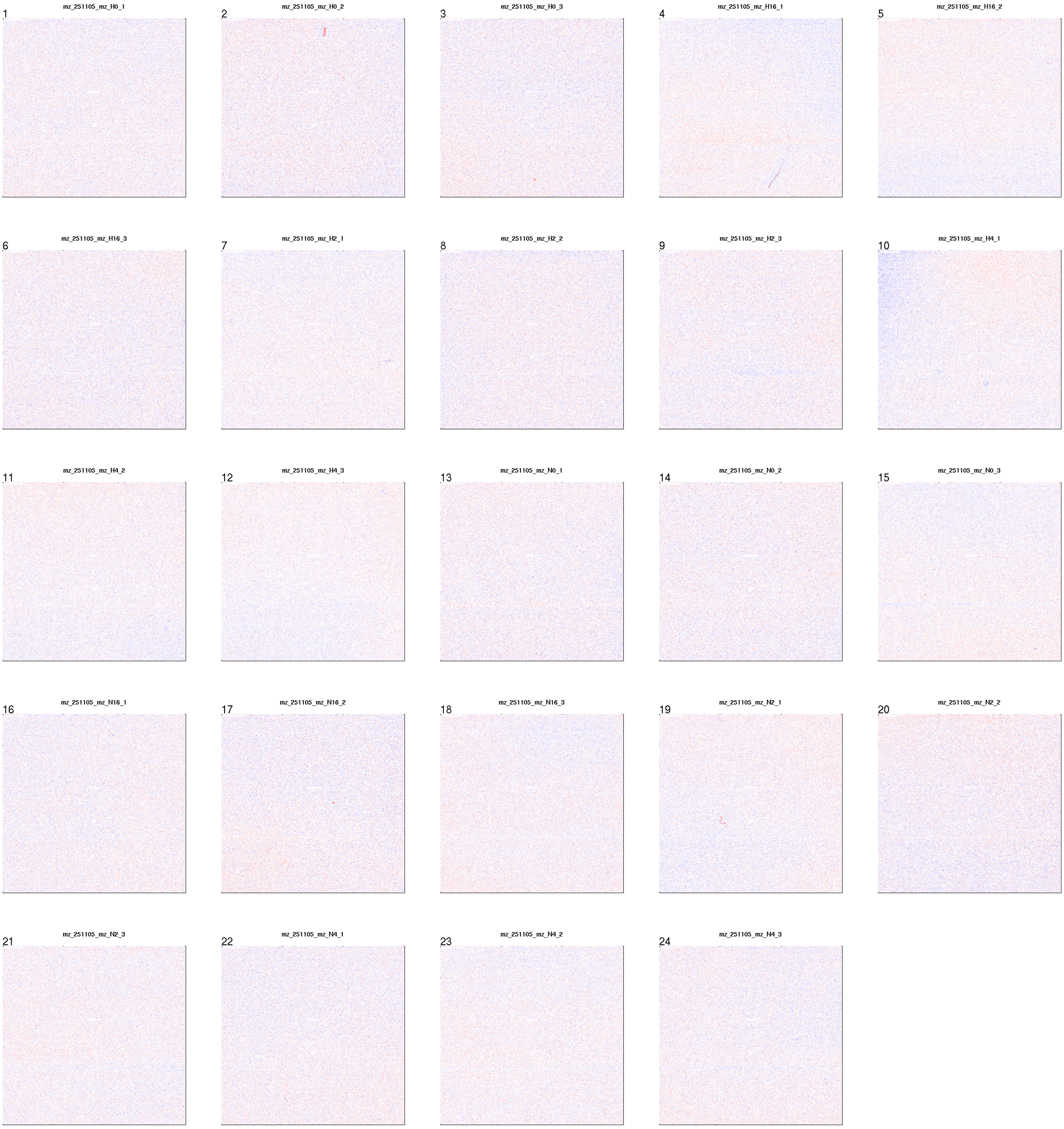
1
mz_251105_mz_H0_1
2
mz_251105_mz_H0_2
3
mz_251105_mz_H0_3
4
mz_251105_mz_H16_1
5
mz_251105_mz_H16_2
6
mz_251105_mz_H16_3
7
mz_251105_mz_H2_1
8
mz_251105_mz_H2_2
9
mz_251105_mz_H2_3
10
mz_251105_mz_H4_1
11
mz_251105_mz_H4_2
12
mz_251105_mz_H4_3
13
mz_251105_mz_N0_1
14
mz_251105_mz_N0_2
15
mz_251105_mz_N0_3
16
mz_251105_mz_N16_1
17
mz_251105_mz_N16_2
18
mz_251105_mz_N16_3
19
mz_251105_mz_N2_1
20
mz_251105_mz_N2_2
21
mz_251105_mz_N2_3
22
mz_251105_mz_N4_1
23
mz_251105_mz_N4_2
24
mz_251105_mz_N4_3

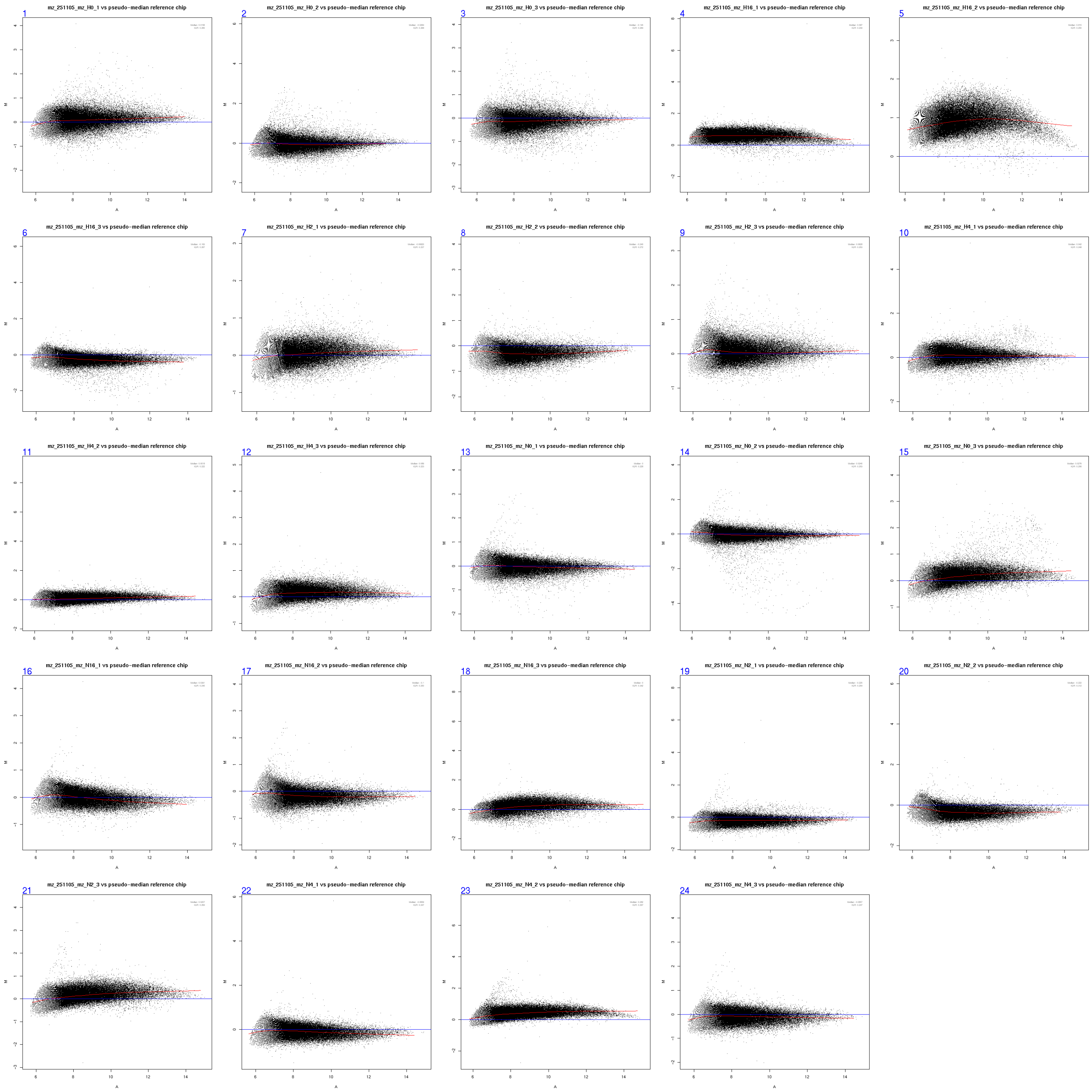

1 mz_251105_mz_H0_1 vs pseudo−median reference chip
2 mz_251105_mz_H0_2 vs pseudo−median reference chip
3 mz_251105_mz_H0_3 vs pseudo−median reference chip
4 mz_251105_mz_H16_1 vs pseudo−median reference chip
5 mz_251105_mz_H16_2 vs pseudo−median reference chip
6 mz_251105_mz_H16_3 vs pseudo−median reference chip
7 mz_251105_mz_H2_1 vs pseudo−median reference chip
8 mz_251105_mz_H2_2 vs pseudo−median reference chip
9 mz_251105_mz_H2_3 vs pseudo−median reference chip
10 mz_251105_mz_H4_1 vs pseudo−median reference chip
11 mz_251105_mz_H4_2 vs pseudo−median reference chip
12 mz_251105_mz_H4_3 vs pseudo−median reference chip
13 mz_251105_mz_N0_1 vs pseudo−median reference chip
14 mz_251105_mz_N0_2 vs pseudo−median reference chip
15 mz_251105_mz_N0_3 vs pseudo−median reference chip
16 mz_251105_mz_N16_1 vs pseudo−median reference chip
17 mz_251105_mz_N16_2 vs pseudo−median reference chip
18 mz_251105_mz_N16_3 vs pseudo−median reference chip
19 mz_251105_mz_N2_1 vs pseudo−median reference chip
20 mz_251105_mz_N2_2 vs pseudo−median reference chip
21 mz_251105_mz_N2_3 vs pseudo−median reference chip
22 mz_251105_mz_N4_1 vs pseudo−median reference chip
23 mz_251105_mz_N4_2 vs pseudo−median reference chip
24 mz_251105_mz_N4_3 vs pseudo−median reference chip

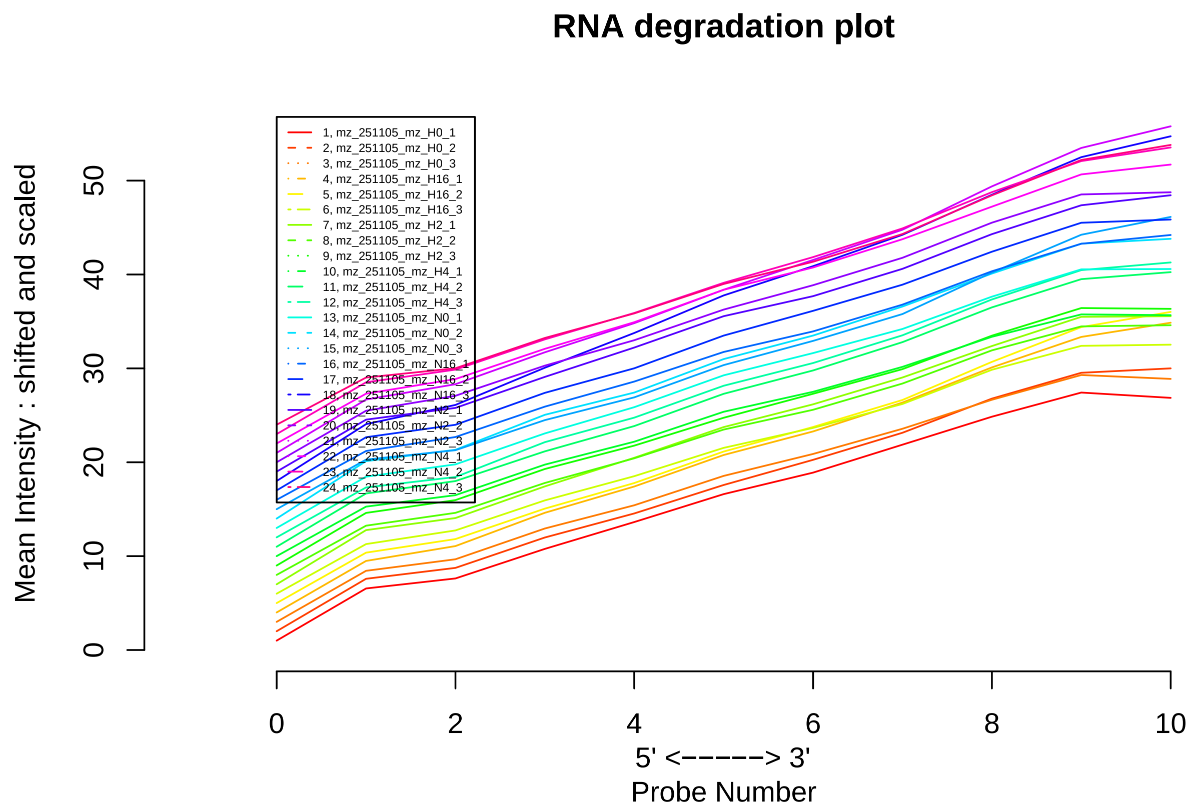
RNA degradation plot
Mean Intensity : shifted and scaled
5' <-----> 3'
Probe Number
1, mz_251105_mz_H0_1
2, mz_251105_mz_H0_2
3, mz_251105_mz_H0_3
4, mz_251105_mz_H16_1
5, mz_251105_mz_H16_2
6, mz_251105_mz_H16_3
7, mz_251105_mz_H2_1
8, mz_251105_mz_H2_2
9, mz_251105_mz_H2_3
10, mz_251105_mz_H4_1
11, mz_251105_mz_H4_2
12, mz_251105_mz_H4_3
13, mz_251105_mz_N0_1
14, mz_251105_mz_N0_2
15, mz_251105_mz_N0_3
16, mz_251105_mz_N16_1
17, mz_251105_mz_N16_2
18, mz_251105_mz_N16_3
19, mz_251105_mz_N2_1
20, mz_251105_mz_N2_2
21, mz_251105_mz_N2_3
22, mz_251105_mz_N4_1
23, mz_251105_mz_N4_2
24, mz_251105_mz_N4_3

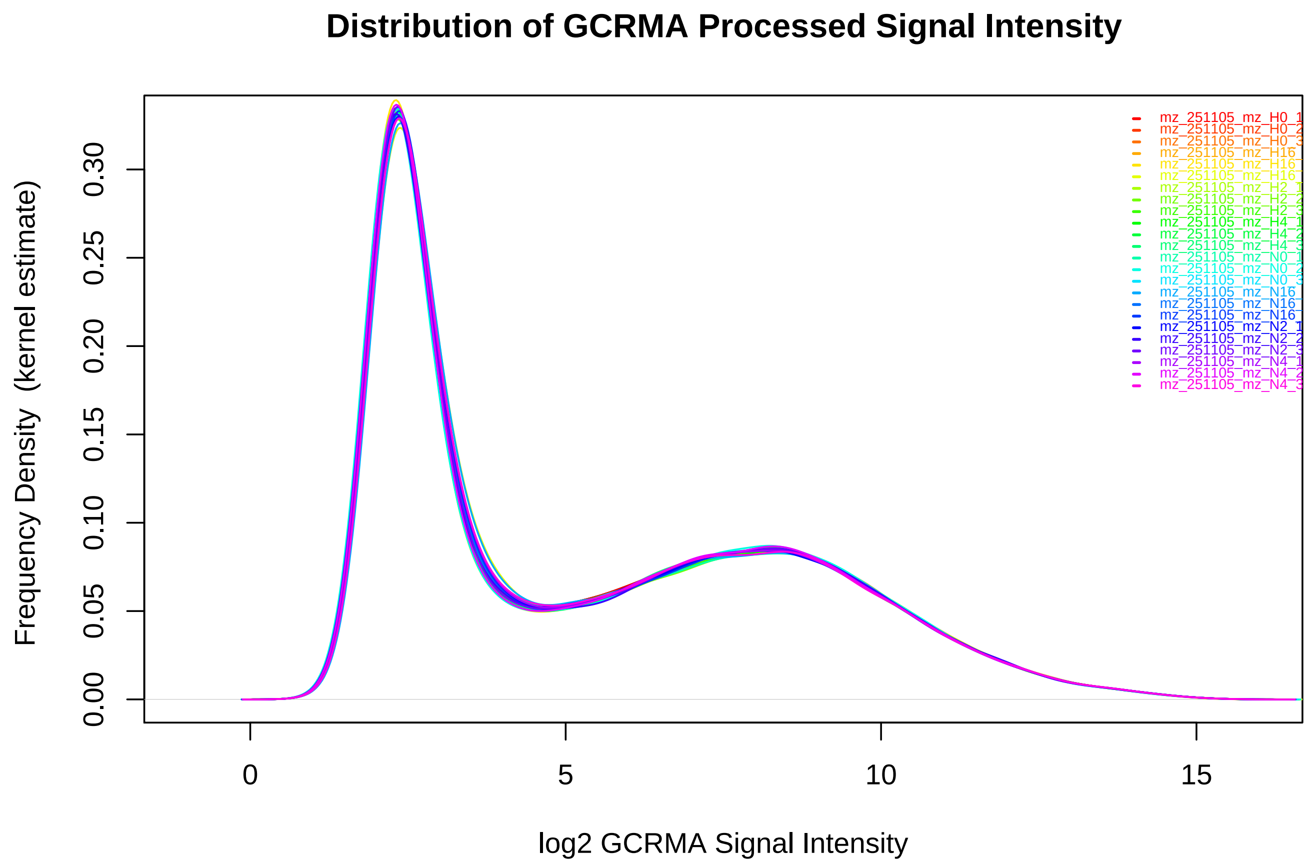
Distribution of GCRMA Processed Signal Intensity
Frequency Density (kernel estimate)
log2 GCRMA Signal Intensity

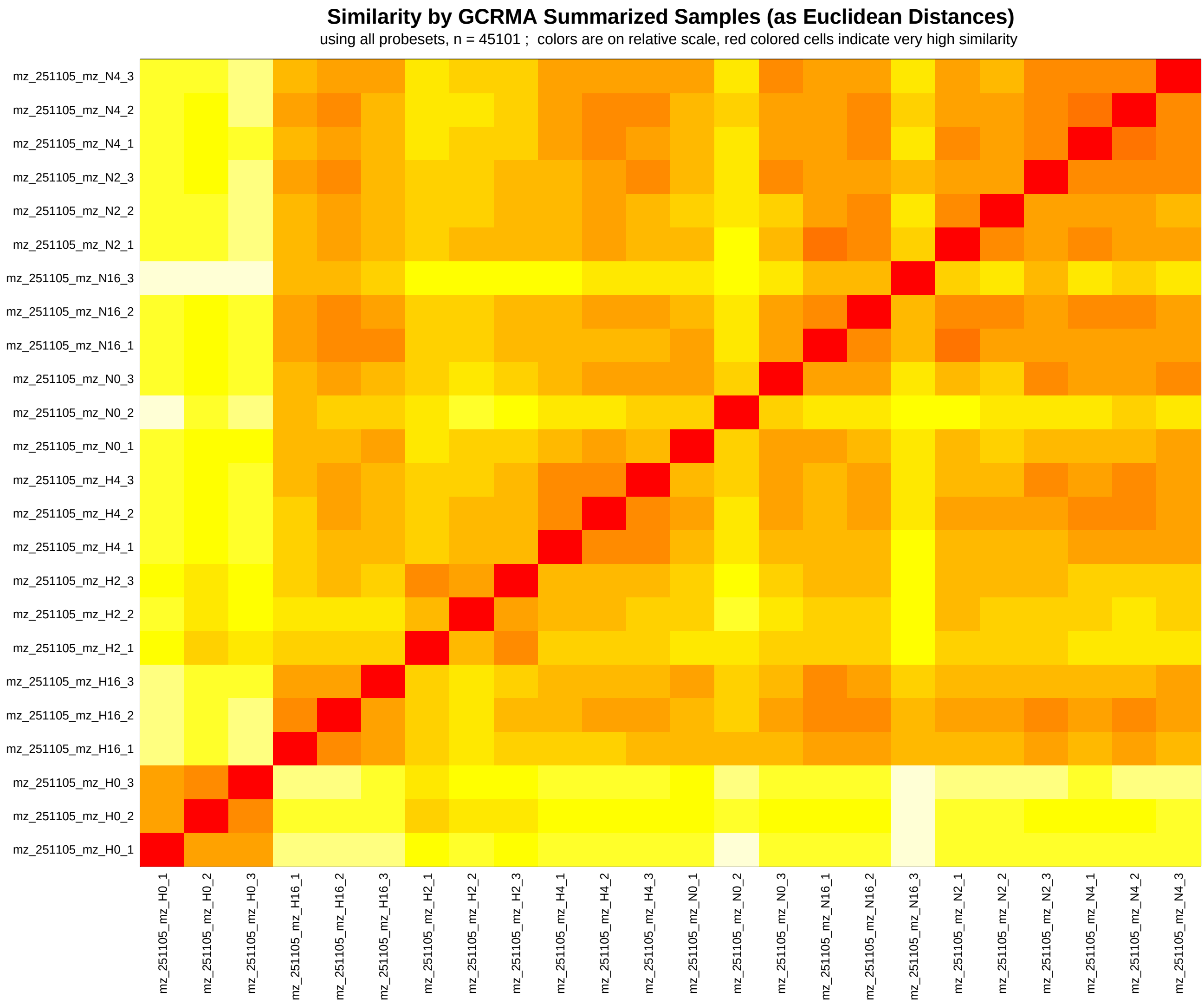
Similarity by GCRMA Summarized Samples (as Euclidean Distances)
using all probesets, n = 45101 ; colors are on relative scale, red colored cells indicate very high similarity
mz_251105_mz_N4_3
mz_251105_mz_N4_2
mz_251105_mz_N4_1
mz_251105_mz_N2_3
mz_251105_mz_N2_2
mz_251105_mz_N2_1
mz_251105_mz_N16_3
mz_251105_mz_N16_2
mz_251105_mz_N16_1
mz_251105_mz_N0_3
mz_251105_mz_N0_2
mz_251105_mz_N0_1
mz_251105_mz_H4_3
mz_251105_mz_H4_2
mz_251105_mz_H4_1
mz_251105_mz_H2_3
mz_251105_mz_H2_2
mz_251105_mz_H2_1
mz_251105_mz_H16_3
mz_251105_mz_H16_2
mz_251105_mz_H16_1
mz_251105_mz_H0_3
mz_251105_mz_H0_2
mz_251105_mz_H0_1

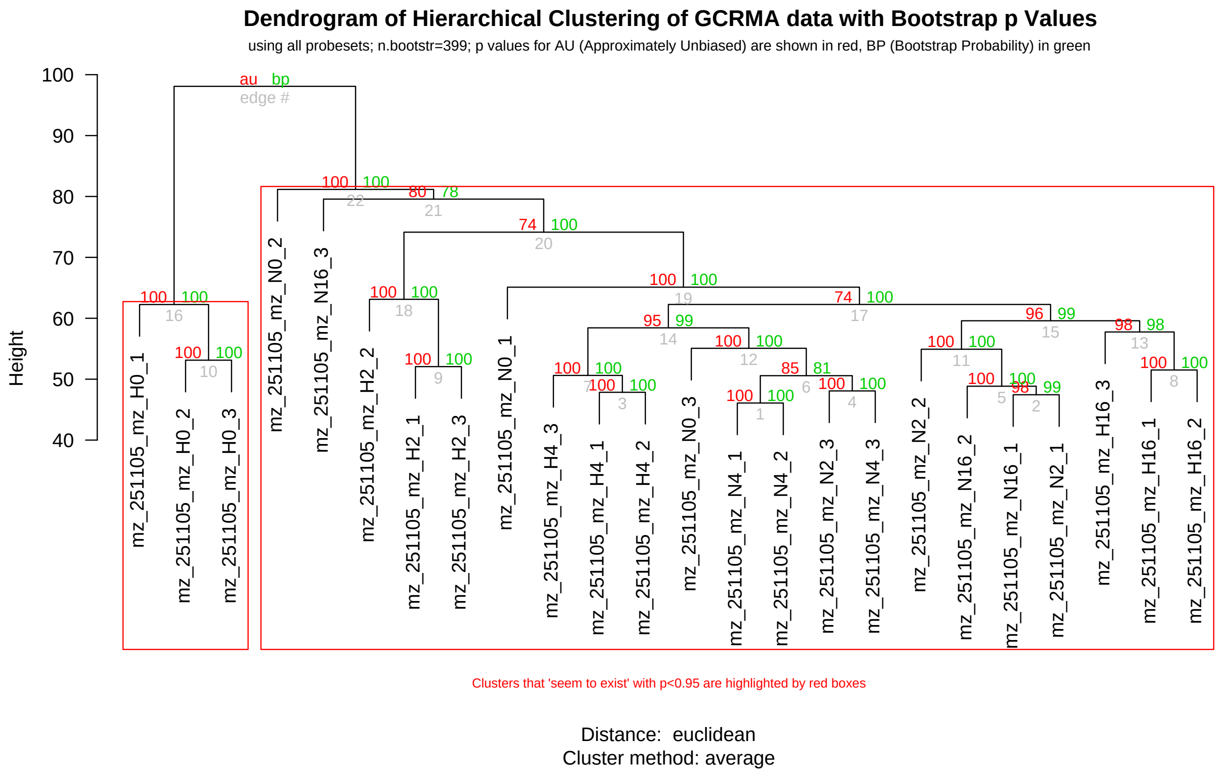
Dendrogram of Hierarchical Clustering of GCRMA data with Bootstrap p Values
using all probesets; n.bootstr=399; p values for AU (Approximately Unbiased) are shown in red, BP (Bootstrap Probability) in green
au bp
edge #
Height
Clusters that 'seem to exist' with p<0.95 are highlighted by red boxes
Distance: euclidean
Cluster method: average

References :

- Raffelsberger W., Krause Y. et al. RReportGenerator : Automatic reports from routine statistical analysis using R. (submitted) and http://www-bio3d-igbmc.u-strasbg.fr/~wraff
- R Development Core Team (2007). R: A language and environment for statistical computing. R Foundation for Statistical Computing, Vienna, Austria. ISBN 3-900051-07-0, http://www.R-project.org.
- Leisch,F. (2002). Sweave: Dynamic generation of statistical reports using literate data analysis. In: Härdle,W. and Rönz,B. (eds), Proceedings in Computational Statistics, Physica Verlag, Heidelberg, pp.575-580
- Gentleman,R.C. et al. (2004) Bioconductor: open software development for computational biology and bioinformatics. Genome Biol., 5, R80.
- Bolstad BM, Irizarry RA, Astrand M, Speed TP. (2003) A comparison of normalization methods for high density oligonucleotide array data based on variance and bias. Bioinformatics. Jan 22;19(2):185-93.
- Irizarry RA, Bolstad BM, Collin F, Cope LM, Hobbs B, Speed TP. (2003) Summaries of Affymetrix GeneChip probe level data. Nucleic Acids Res. 2003 Feb 15;31(4):e15.
- Gautier L, Cope L, Bolstad BM, Irizarry RA. (2004) affy–analysis of Affymetrix GeneChip data at the probe level. Bioinformatics. 2004 Feb 12;20(3):307-15.
- Rafael A. Irizarry, Laurent Gautier, Benjamin Milo Bolstad, and Crispin Miller with contributions from Magnus Astrand, Leslie M. Cope, Robert Gentleman, Jeff Gentry, Conrad Halling, Wolfgang Huber, James MacDonald, Benjamin I.P. Rubinstein, Christopher Workman and John Zhang (). affy: Methods for Affymetrix Oligonucleotide Arrays. R package.
- Wilson C.L., Miller C.J. (2005) Simpleaffy: a BioConductor package for Affymetrix Quality Control and data analysis. Bioinformatics. 2005 Sep 15;21(18):3683-5.
- Ben Bolstad (2007). affyPLM: Methods for fitting probe-level models. R package. http://bmbolstad.com
- Craig Parman, Conrad Halling and Robert Gentleman (). affyQCReport: QC Report Generation for affyBatch objects. R package
- Reimers M, Weinstein JN. (2005) Quality assessment of microarrays: visualization of spatial artifacts and quantitation of regional biases. BMC Bioinformatics. 2005 Jul 1;6:166.
- Smyth, G. K. (2005). Limma: linear models for microarray data. In: 'Bioinformatics and Computational Biology Solutions using R and Bioconductor'. R. Gentleman, V. Carey, S. Dudoit, R. Irizarry, W. Huber (eds), Springer, New York, pages 397–420.
- Suzuki R, Shimodaira H. (2004) Pvclust: an R package for assessing the uncertainty in hierarchical clustering. Bioinformatics. Jun 15;22(12):1540-2.
- http://www.affymetrix.com

time for reading cel-fiels : 212.46 sec
time for figures QC1 and QC2 : 320.76 sec
time for RNA degradation figure : 25.13 sec
time for plm calculation : 72.62 sec
time for RLE and NUSE figures : 14.75 sec
time for residual images : 603.45 sec
time for MA-plots : 800.85 sec
time for calculating RMA : 252.31 sec
time for plot of Sig Dist of RMA treated data : 2.08 sec
time for hierarchical clustering and bootstrap pValues : 138.9 min
