# Supplementary material for: Analysis of the retinal gene expression profile after hypoxic preconditioning identifies candidate genes for neuroprotection
Source: BMC Genomics. 2008 Feb 8;9:73. doi: 10.1186/1471-2164-9-73 (PMC2270833; doi:10.1186/1471-2164-9-73)
Supplement: Additional file 3 — Table 4 Real time PCR results. Table 4 Real-Time PCR results of the expression of genes detected by Affymetrix microarrays as differentially regulated by hypoxic preconditioning. File contains all genes revealed by microarray analyses, which were tested by real time PCR. [file 1471-2164-9-73-S3.doc]

TABLE 4 *Real-Time PCR results of genes detected by Affymetrix microarrays as differentially regulated by hypoxic preconditioning*.

| **Gene Symbol** | **Gene** | **H0 Chip** | **HO**  **RT** | **pVal** | **H2 Chip** | **H2**  **RT** | **pVal** | **H4 Chip** | **H4**  **RT** | **pVal** | **H16 Chip** | **H16 RT** | **pVal** |
| --- | --- | --- | --- | --- | --- | --- | --- | --- | --- | --- | --- | --- | --- |
| Adm | adrenomedullin | 10.59 | 9.36 | *** | - | 2.5 | ** | - | 1.21 | n.s. | - | 1.05 | n.s. |
| Bcl2l10 | Bcl2-like 10 | 17.02 | 30.44 | *** | - | n.a. |  | - | n.a. |  | - | n.a. |  |
| Cdkn1a (p21) | Cyclin dependent kinase inhibitor 1a | 30.48 | 20.26 | ** | - | 4.64 | ** | - | 1.51 | n.s. | - | 0.8 | n.s. |
| CEBP/d | CCAAT/enhancer binding protein (C/EBP), delta | 3.47 | 3.47 | * | - | n.a. |  | - | n.a. |  | - | n.a. |  |
| Dido1 (Iso1/3) | death inducer-obliterator 1 | 0.3 | 0.86 | n.s. | - | n.a. |  | - | n.a. |  | - | n.a. |  |
| Dido1 (Iso2) | death inducer-obliterator 1 | 0.3 | 1.21 | n.s. | - | n.a. |  | - | n.a. |  | - | n.a. |  |
| Egf | epidermal growth factor | 4.7 | 2.46 | ** | - | 1.03 | n.s. | - | 1 | n.s. | - | 1.71 | * |
| Egln1 | Egl nine homolog 1 (C. elegans) | 4.44 | 5.01 | ** | - | n.a. |  | - | n.a. |  | - | n.a. |  |
| Fabp4 | Fatty acid binding protein 4, adipocyte (Fabp4), mRNA | 3.5 | 5.33 | * | - | n.a. |  | - | n.a. |  | - | n.a. |  |
| H3f3B | H3 histone, family 3B | 6.28 | 1.58 | n.s. | - | n.a. |  | - | n.a. |  | - | n.a. |  |
| Hes6 | hairy and enhancer of split 6 (Drosophila) | 1.87 | 1.59 | * | - | n.a. |  | - | n.a. |  | - | n.a. |  |
| Hmgb2 | high mobility group box 2 | 4.06 | 3.05 | n.s. | - | n.a. |  | - | n.a. |  | - | n.a. |  |
| Ibrdc2 | IBR domain containing 2 (Ibrdc2), mRNA | 0.29 | 0.96 | n.s. | - | n.a. |  | - | n.a. |  | - | n.a. |  |
| Id1 | inhibitor of DNA binding 1 | 3.1 | 1.75 | ** | - | n.a. |  | - | n.a. |  | - | n.a. |  |
| Kif4 | kinesin family member 4A | - | 2.94 | ** | 8.91 | 4.42 | ** | 13.94 | 6.32 | *** | - | 1.31 | n.s. |
| Mef2c | myocyte enhancer factor 2C | 0.31 | 0.7 | * | - | n.a. |  | - | n.a. |  | - | n.a. |  |
| Metap2 | methionine aminopeptidase 2 | 2.74 | 0.81 | n.s. | - | 0.95 | n.s. | - | 1.18 | n.s. | - | 0.95 | n.s. |
| Mt1 | metallothionein 1 | 6.26 | 6.89 | *** | 6.11 | n.a. |  | - | n.a. |  | - | n.a. |  |
| Pon1 | paraoxonase 1 | 22.55 | 7.6 | *** | - | 1.3 | n.s. | - | 1.7 | n.s. | - | 0.7 | n.s. |
| Rad23b | RAD23b homolog (S. cerevisiae) | 2.6 | 1.17 | n.s. | - | n.a. |  | - | n.a. |  | - | n.a. |  |
| Sema3c | Semaphoring 3c | 5.86 | 3.86 | * | - | n.a. |  | - | n.a. |  | - | n.a. |  |
| Slc2A1 (Glut1) | solute carrier family 2 (facilitated glucose transporter), member 1 | 1.98 | 1.73 | *** | - | n.a. |  | - | n.a. |  | - | n.a. |  |
| Sos1 | Son of sevenless homolog 1 (Drosophila) | 1.92 | 0.88 | n.s. | - | n.a. |  | - | n.a. |  | - | n.a. |  |
| Stat1 | signal transducer and activator of transcription 1 | 0.47 | 1.11 | n.s. | - | n.a. |  | - | n.a. |  | - | n.a. |  |
| Stom | stomatin | 2.03 | 1.29 | n.s. | - | n.a. |  | - | n.a. |  | - | n.a. |  |
| Thra | thyroid hormone receptor alpha | 0.48 | 0.78 | n.s. | - | n.a. |  | - | n.a. |  | - | n.a. |  |
| Timp3 | tissue inhibitor of metalloproteinase 3 | 3.28 | 2.35 | *** | - | 1.45 | n.s. | - | 1.24 | n.s. | - | 1.64 | * |
| Ttr | transthyretin | 20.11 | 8.8 | ** | - | 1.2 | n.s. | - | 1.7 | n.s. | - | 0.5 | n.s. |
| Vegfa_all | vascular endothelial growth factor | 2.25 | 2 | * | - | 0.6 | * | - | 1.1 | n.s. | - | 1.5 | ** |
| Vegf164 (Iso1) | vascular endothelial growth factor isoform1 | 2.25 | 2.5 | * | - | 0.7 | n.s. | - | 1.3 | n.s. | - | 1.1 | n.s. |
| Vegf120 (Iso2) | vascular endothelial growth factor isoform2 | 2.25 | 1.9 | * | - | 1.1 | n.s. | - | 0.9 | n.s. | - | 1.3 | n.s. |
| Vegf188 (Iso3) | vascular endothelial growth factor isofrom 3 | 2.25 | 3 | * | - | 0.9 | n.s. | - | 1 | n.s. | - | 0.9 | n.s. |

n.a. not analyzed, n.s. not significant, (* p ≤ 0.05; ** p ≤ 0.01; *** p ≤ 0.001)
