# Supplementary material for: Analysis of the retinal gene expression profile after hypoxic preconditioning identifies candidate genes for neuroprotection
Source: BMC Genomics. 2008 Feb 8;9:73. doi: 10.1186/1471-2164-9-73 (PMC2270833; doi:10.1186/1471-2164-9-73)

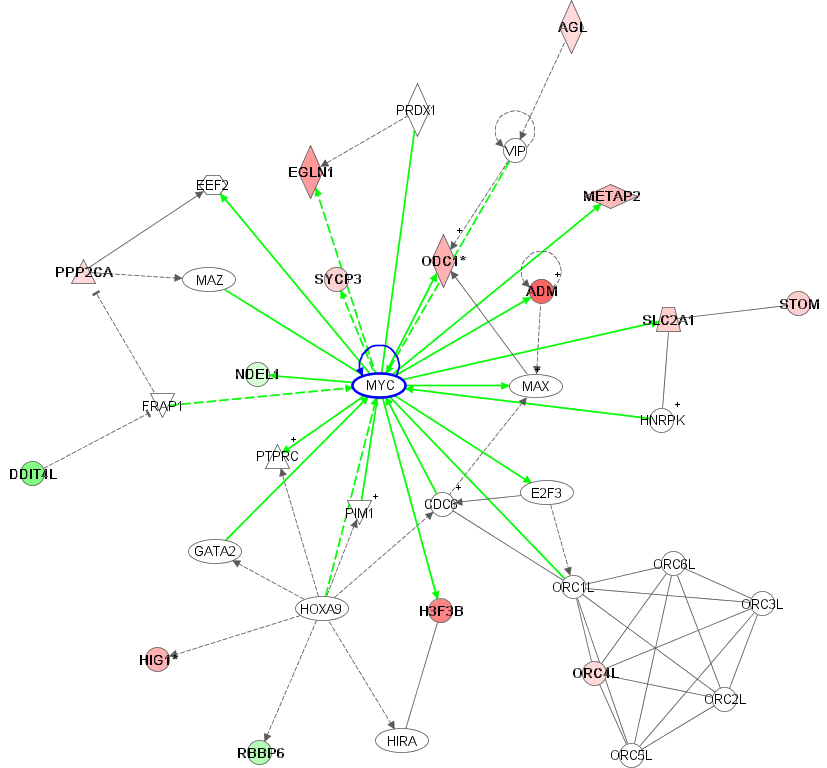


Fig.2 A prominent affected gene network discovered by Ingenuity Pathway Analysis. Network was classified as: *DNA replication, recombination, cell cycle and cancer*. Pathway is centrally occupied by Myc and contains the pro survival gene Adm. Note that Myc itself is not differentially regulated. (*red = induction; green = repression; white = unaffected; color intensity correlates with fold change*)


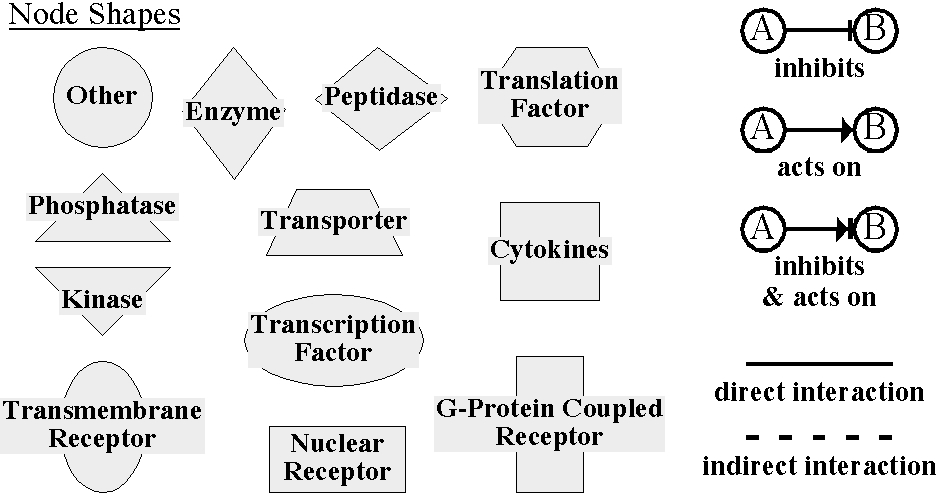


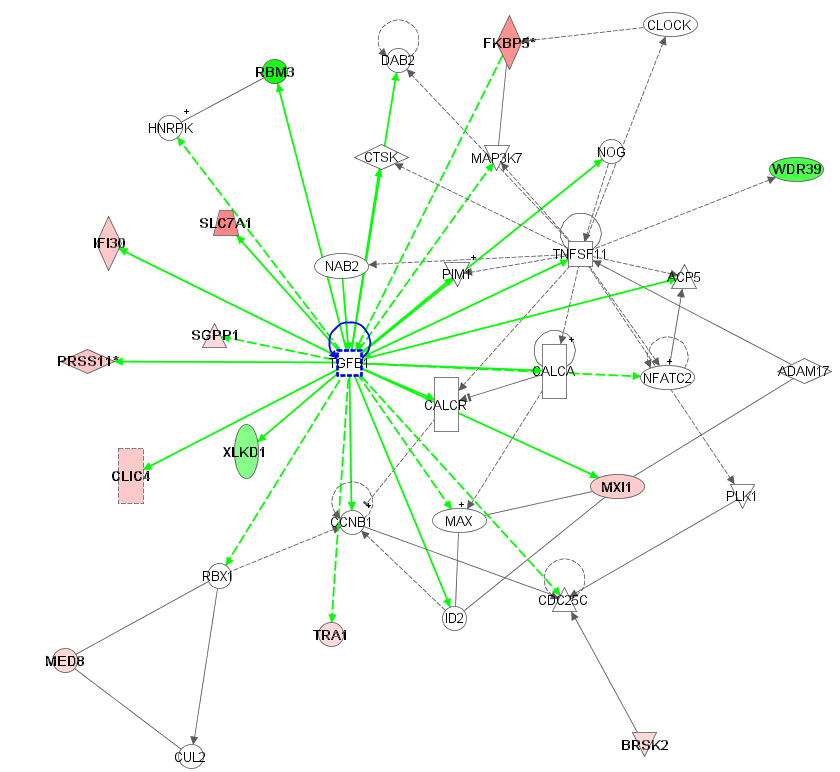


Fig.3 An affected gene network discovered by Ingenuity Pathway Analysis. Network was classified as: *Cell death, cellular development, hematological system development & function*. Some genes related to TGF-β are induced after hypoxic preconditioning (*red = Induction; green = repression; white = unaffected; color intensity correlates with fold change*)


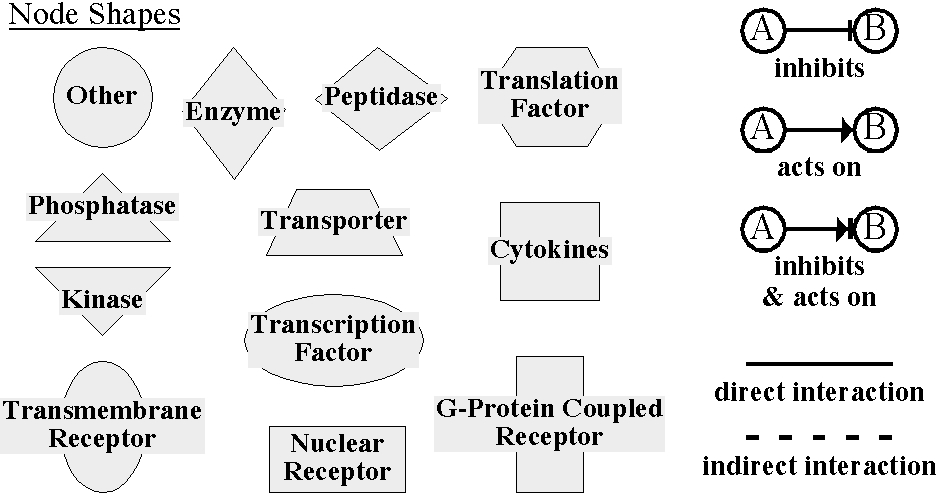

Supplement: Additional file 5 — Fig 2, 3 Hypoxic preconditioning gene networks discovered by Ingenuity Pathway analysis. Fig 2 affected gene network discovered by Ingenuity Pathway Analysis and classified as: DNA replication, recombination, cell cycle and cancer. Fig. 3 affected gene network discovered by Ingenuity Pathway Analysis and classified as: Cell death, cellular development, hematological system development & function. File contains schemes of pathways, which are affected by hypoxic preconditioning. Some genes connected to cMyc (Fig. 7) and to TGF-b (Fig. 8) are differentially regulated. [file 1471-2164-9-73-S5.doc]
