# Supplementary material for: Analysis of the retinal gene expression profile after hypoxic preconditioning identifies candidate genes for neuroprotection
Source: BMC Genomics. 2008 Feb 8;9:73. doi: 10.1186/1471-2164-9-73 (PMC2270833; doi:10.1186/1471-2164-9-73)
Supplement: Additional file 6 — Table 7 Primer Sequences and product sizes. Table 7 Primer pairs used for real time PCR. File contains sequences of Primers used for real time PCR and the expected/obtained product sizes in bp. [file 1471-2164-9-73-S6.doc]

TABLE 7 *Primer pairs used for real time PCR.*

| Gene | Primer forward | Primer reverse | Product bp |
| --- | --- | --- | --- |
| Adm | ttcgcagttccgaaagaagt | ggtagctgctggatgcttgta | 77 |
| Bcl2l10 | GAACTTTCTGTATAATCTGCTCATGG | TGAAGAAGCGGCAAAAGC | 89 |
| Cdkn1a | CGGTGTCAGAGTCTAGGGGAATTG | CGTGACGAAGTCAAAGTTCCACC | 238 |
| CEBPd | cttttaggtggttgccgaag | gcaacgaggaatcaagtttca | 70 |
| Dido1 (Iso1/3) | ctctatatccgtggtagctctgg | tggggactgcctcttaaaca | 76 |
| Dido1 (Iso2) | gactgagttcgaaacgaagga | cgaacaaccaaagggacagt | 63 |
| Egf | catgccccacaggatttg | gggcaggaaacaagttcgt | 64 |
| Egln1 | cattgttggcagaaggtgtg | caaaggactacagggtctcca | 70 |
| Fabp4 | gaaaacgagatggtgacaagc | ttgtggaagtcacgccttt | 60 |
| H3f3B | cgattgcggctcttgttc | cttggttcgggccatttt | 96 |
| Hes6 | gcacggatcaacgagagtc | cggcgttctctagcttgg | 76 |
| Hmgb2 | gattgcgttacgagaaaccag | gtcacccttgcccatgac | 126 |
| Ibrdc2 | aggctgtggctcagacctta | acactggagcccatcgac | 75 |
| Id1 | gcgagatcagtgccttgg | ctcctgaagggctggagtc | 111 |
| Kif4 | gcatgactgcaaccattgat | tatctgggctgctttgcact | 63 |
| Mef2c | tctgccctcagtcagttgg | cgtggtgtgttgtgggtatc | 63 |
| Metap2 | ggagagagatgacgacgatga | cttcttcccagttgcaccat | 61 |
| Mt1 | gaatggaccccaactgctc | gcagcagctcttcttgcag | 104 |
| Pon1 | AATGCTTTCCGTGAAGTAACGC | TCTAACTCTGACACTGCTGGCTCC | 218 |
| Rad23b | ccctgacagagctgtggaat | gtcaaccacagcctgactttc | 70 |
| Sema3c | atggccactcttgctctagg | catcttgtcttcggctcctc | 60 |
| Slc2A1 | atggatcccagcagcaag | ccagtgttatagccgaactgc | 92 |
| SOS1 | tcggcaactcactttacttgaa | tgtccacacacttccaactaattc | 76 |
| Stat1 | ttgtgttgaatcccgaacct | tcgaaccactgtgacatcct | 95 |
| Stom | ccagtgcagctccagagag | cgcattcatttccccttc | 93 |
| Thra | aaggtggagtgtgggtcaga | tttttcgctttccatctggt | 64 |
| Timp3 | gcctcaagctagaagtcaacaaa | tgtacatcttgccttcatacacg | 69 |
| Ttr | AATGTCCTCTGATGGTCAAAGTCC | TGGAACGGGGAAATGCCAAG | 238 |
| Vegf_all | ACTTGTGTTGGGAGGAGGATGTC | AATGGGTTTGTCGTGTTTCTGG | 171 |
| Vegf164 (Iso1) | CATCTTCAAGCCGTCCTGTGTG | TGACCCTTTCCCTTTCCTCG | 240 |
| Vegf120 (Iso2) | GCCAGAAAATCACTGTGAGCC | TCTACAGGAATCCCAGAAACAACC | 478 |
| Vegf188 (Iso3) | CAGAAAAATGTGACAAGCCAAGG | TCTACAGGAATCCCAGAAACAACC | 344 |
